# Machine Learning Prediction of 90-Day Readmission and Mortality in General Internal Medicine: A Retrospective Cohort Study

**DOI:** 10.3390/diagnostics16203337

**Published:** 2026-10-14

**Authors:** Laila Al Wahaibi, Said Al Jaadi, Mohammed Al-Hinai, Abdullah M. Al Alawi

**Affiliations:** 1 Internal Medicine Residency Program, Oman Medical Specialty Board, Muscat 123, Omanr23070@resident.omsb.org (S.A.J.); 2 College of Medicine and Health Sciences, Sultan Qaboos University, Muscat 123, Oman; 3 Medical Research Center, Sultan Qaboos University, Muscat 123, Oman; 4 Department of Medicine, Sultan Qaboos University Hospital, Muscat 123, Oman

**Keywords:** machine learning, readmission, post-discharge mortality, general internal medicine, risk stratification, electronic health record (EHR), SMOTE, AI

## Abstract

**Background/Objectives:** Predicting post-discharge outcomes in general internal medicine (GIM) is essential for targeted intervention. This study developed machine learning (ML) models to predict 90-day hospital readmission and 90-day all-cause post-discharge mortality using routine clinical data. **Methods:** This retrospective cohort study included 935 adult admissions to a tertiary GIM department (January 2022–December 2023), of which 881 were retained after data quality verification. Six ML algorithms—Logistic Regression, Random Forest, Gradient Boosting, Support Vector Machine (SVM), Neural Network, and Voting Ensemble—were evaluated across admission-only (Tier 1) and discharge-augmented (Tier 2) feature sets using Synthetic Minority Over-sampling Technique (SMOTE), SelectKBest feature selection, and five-fold cross-validated hyperparameter tuning. **Results:** The 90-day readmission and mortality rates were 33.4% and 4.8%, respectively. Mortality models substantially outperformed readmission models (best admission-model AUC 0.821 for the Voting Ensemble vs. 0.645 for Logistic Regression in readmission; best discharge-model mortality AUC 0.828 for Logistic Regression, 95% CI: 0.694–0.930). Discharge models did not improve over admission models for either outcome. Key mortality predictors included code status, albumin level, functional dependence, and age. **Conclusions:** Admission-available clinical variables enabled good discrimination of 90-day mortality but only modest readmission prediction. Given the low events-per-variable ratio and single-centre design, these findings should be considered hypothesis-generating and require external validation before clinical implementation.

## 1. Introduction

Unplanned hospital readmission and post-discharge mortality represent two of the most consequential and costly outcomes in general internal medicine [1]. In most healthcare systems, readmission rate ranges from about 16% to 27% within 30 days, while post-discharge mortality contributes substantially to avoidable deaths in the weeks and months following hospitalization [2,3,4,5,6]. Beyond their clinical significance, these outcomes impose a considerable burden on healthcare resources, occupying hospital beds, straining staffing capacity and generating substantial costs that are increasingly subject to regulatory and financial scrutiny. Despite their recognized importance, we still lack systematic methods to identify high-risk patients at discharge [7].

General internal medicine wards present a particularly challenging environment for risk stratification. A heterogeneous population with a broad spectrum of acute and chronic conditions, multimorbidity, functional decline, and social complexity is admitted to general internal medicine wards, resulting in highly diverse clinical profiles that resist uniform risk assessment [5,8]. Traditional scoring systems such as APACHE and SAPS were developed for ICU patients and have well-recognized limitations when applied to unselected general medicine cohorts [9,10]. Similarly, established comorbidity indices including the Charlson Comorbidity Index (CCI), Comprehensive Geriatric Assessment (CGA) and Geriatric Index of Comorbidity (GIC) demonstrate variable and often modest predictive performance for post-discharge outcomes, reflecting their inability to integrate the full complexity of clinical data routinely available at the bedside [11,12,13]. Furthermore, across multiple settings, clinician-based mortality predictions show only moderate accuracy and substantial variability and are often miscalibrated so combining them with objective reproducible risk models improves prognostic performance and supports more consistent risk stratification [14,15,16].

Combined techniques such as Random Forest and Gradient Boosting, support vector machines, and neural network architectures have all demonstrated strong discriminatory performance across a range of clinical prediction tasks, including in-hospital mortality, sepsis, acute kidney injury, and readmission [17,18,19,20,21].

An expanding body of literature has applied ML to the prediction of hospital readmission and post-discharge mortality, with encouraging outcomes in a variety of clinical contexts [22,23,24]. However, several important gaps remain. The majority of published models target either in-hospital outcomes or disease-specific populations such as heart failure, COPD, or pneumonia, with relatively few studies examining post-discharge outcomes in unselected general medicine cohorts [22,25,26,27]. Additionally, most existing studies evaluate a single outcome using a single algorithm, limiting cross-outcome and cross-model comparisons that would be valuable for informing clinical implementation decisions. A further underexplored question is whether clinical information accrued during hospitalization including vital sign trajectories, length of stay, and hospital-acquired complications provides meaningful incremental predictive value over variables available at the time of admission. Addressing this question has direct clinical implications, as admission-time prediction would allow earlier identification and intervention for at-risk patients [5].

The present study is embedded within a broader research programme examining the utility of ML for clinical prediction in general internal medicine. A companion study previously applied ML models to predict inpatient clinical deterioration defined as unplanned intensive care unit (ICU) transfer or in-hospital death in a subset of 524 patients from the same institution, where a Random Forest model achieved an area under the receiver operating characteristic curve (AUC) of 0.837 [28]. The present work extends this programme by utilizing the full cohort of 935 patients and shifting the predictive focus to post-discharge outcomes, thereby addressing a complementary and clinically significant gap in the care continuum.

This study develops and evaluates machine learning models using routine EHR data to predict 90-day hospital readmission and all-cause post-discharge mortality in general internal medicine patients. It also compares six algorithms, assesses admission versus discharge feature strategies, identifies key predictors, and characterizes baseline differences between patients with and without each outcome to support ML-assisted discharge risk stratification.

## 2. Materials and Methods

### 2.1. Study Design and Population

This retrospective cohort study utilized data from 935 adult patients admitted to the General Internal Medicine (GIM) department of a tertiary care hospital between January 2022 and December 2023. The dataset, originally compiled for a published study on inpatient clinical deterioration prediction, ref. [28] was repurposed to develop machine learning models for predicting two post-discharge outcomes: 90-day hospital readmission and 90-day all-cause mortality. This study was conducted in accordance with the Transparent Reporting of a multivariable prediction model for Individual Prognosis or Diagnosis (TRIPOD) guidelines for prediction model development [29].

A subset of 524 patients from this dataset was previously analyzed in a companion study focused on predicting inpatient deterioration [28]. The present study uses the full cohort of 935 patients and targets post-discharge outcomes.21§

### 2.2. Data Quality Assurance and Outcome Verification

A date-based quality audit verified all 935 records prior to analysis. Length of stay was independently computed from admission and discharge dates, confirming 100% concordance. Outcome classifications were verified by computing discharge-to-event intervals. This audit identified 38 readmission reclassifications (23 patients originally flagged as readmitted but with dates exceeding 90 days; 15 patients not flagged but with documented readmission within 90 days) and 7 mortality reclassifications (3 flagged but outside the 1–90 day window; 4 not flagged but with death dates within 1–90 days of discharge). All were corrected prior to modelling.

### 2.3. Study Population and Exclusion Criteria

From the initial 935 patients, the following were excluded: patients who died during the index hospitalization, as verified by date-based triangulation; patients with death dates preceding their admission date, indicating data entry errors; patients with a negative calculated age; patients with readmission dates preceding their discharge date; and patients with missing readmission outcome flags; patients with missing readmission outcome flags. These exclusions were not mutually exclusive, yielding a final analyzable cohort of 881 patients.

### 2.4. Outcome Definitions

Two primary outcomes were analyzed as separate binary classification targets. The first outcome, 90-day hospital readmission, was defined as any emergency department presentation or unplanned hospital readmission within 90 days of discharge. This was determined using a pragmatic, date-first approach: patients with documented readmission dates within 0 to 90 days of discharge were classified as readmitted regardless of the original flag; patients flagged as readmitted but without a documented date were excluded from the analysis (*n* = 2), as these could not be independently verified. The date-first approach was adopted because documented dates were considered more reliable than retrospective clinical flags, and patients with documented readmission dates exceeding 90 days were reclassified as not readmitted. The second outcome, 90-day post-discharge all-cause mortality, was defined as death occurring between 1 and 90 days after hospital discharge, verified by the interval between the discharge date and the documented date of death. Data were collected through review of hospital electronic records and supplemented with phone follow-up calls.

### 2.5. Predictor Variables and Feature Engineering

A two-tiered feature strategy was employed to evaluate the predictive value of information available at different clinical time points. The admission model (Tier 1) utilized only variables available within eight hours of hospital admission, including demographics (age, sex, body mass index), admission context (day of admission, functional status, code status), prior healthcare utilization (admissions in the preceding 3 and 12 months, emergency department presentations in the preceding 12 months), comorbidities (diabetes mellitus, hypertension, dyslipidemia, cardiovascular disease, heart failure, chronic obstructive pulmonary disease, cerebrovascular disease, active malignancy, dementia, acute kidney injury, chronic kidney disease, liver cirrhosis, and a composite indicator of three or more comorbidities), medications (polypharmacy, antiepileptics, diuretics, anticoagulants), social history (smoking, alcohol use, substance use), admission vital signs within eight hours (temperature, heart rate, respiratory rate, systolic and diastolic blood pressure), oxygen requirements, and admission laboratory values (hemoglobin, white blood cell count, platelets, creatinine, sodium, potassium, urea, albumin, C-reactive protein, anion gap, bicarbonate, chloride, estimated glomerular filtration rate, and calcium). Admission diagnoses were grouped into ten categories based on ICD-10 coding: respiratory, cardiovascular, cerebrovascular, infectious, gastrointestinal, metabolic, hematological, viral, renal, and other.

The discharge model (Tier 2) incorporated all Tier 1 features plus variables recorded during the hospitalization, including length of stay, vital signs at 16 and 24 h, delta features representing temporal change in vital signs (heart rate, systolic blood pressure, temperature, and respiratory rate change from 8 to 24 h), hospital-acquired infection, and blood transfusion during admission.

### 2.6. Missing Data Handling

Variables with greater than 15% missing values were excluded from the analysis, consistent with the approach used in the companion inpatient deterioration study [28]. Excluded variables included troponin (37% missing), arterial pH (29%), glucose (31%), lactate (33%), lactate dehydrogenase (79%), brain natriuretic peptide (82%), ferritin (82%), vitamin B12 (53%), and international normalized ratio (24%). An exception was made for body mass index (27% missing), which was retained given its established clinical relevance in post-discharge outcome prediction. Missing values in retained variables were imputed using median imputation for continuous variables and mode imputation for categorical variables. Among the 52 retained features, 25 had some missing values; the highest missingness rates were AST (14.8%), calcium (10.2%), CRP (10.0%), and ALT (9.1%), with most variables showing less than 5% missingness, consistent with a missing-at-random pattern typical of routine clinical data. Median imputation was chosen for its simplicity, robustness to outliers, and consistency with the companion study [28]. More sophisticated methods such as multiple imputation (MICE) may better preserve variance structure but were not employed given the low overall missingness and the exploratory nature of this study.

### 2.7. Feature Selection

Feature selection was performed using SelectKBest with the analysis of variance (ANOVA) F-test statistic, applied exclusively to the training set after the train–test split to prevent data leakage. The feature mask derived from training data was then applied to transform the held-out test set. Based on the differing event rates, 20 features (k = 20) were selected for readmission models and 10 features (k = 10) for mortality models. The lower value for mortality was chosen to maintain an appropriate events-per-variable (EPV) ratio; with 42 mortality events in the full cohort, k = 10 yields an EPV of 4.2, already below the recommended minimum of 10–20 for reliable prediction model development [30]. A sensitivity analysis with k = 5 (EPV = 8.4) was also conducted. A LASSO-based feature selection comparison confirmed substantial overlap between univariate and multivariate feature rankings (7/10 features shared for mortality).

### 2.8. Class Imbalance Handling

The Synthetic Minority Over-sampling Technique (SMOTE) was applied exclusively to the training set to address class imbalance, particularly for the mortality outcome (4.8% event rate). SMOTE generates synthetic minority class examples by interpolating between existing minority instances and their nearest neighbours. The technique was applied only after the train–test split to prevent data leakage from the test set. For the mortality outcome, approximately 95% of minority-class training examples were synthetic (558 synthetic vs. 29 original minority instances), reflecting the severity of the class imbalance. As a sensitivity analysis, class weighting (balanced) was also evaluated as an alternative to SMOTE, and a no-balancing condition was tested (Supplementary Table S1).

### 2.9. Machine Learning Models

Six machine learning algorithms were evaluated: Logistic Regression, Random Forest, Gradient Boosting, Support Vector Machine (SVM) with radial basis function kernel, Multi-layer Perceptron Neural Network, and a Voting Classifier ensemble. Logistic Regression served as the baseline interpretable model. Random Forest and Gradient Boosting represented ensemble tree-based methods. The SVM model used a radial basis function kernel with probability estimation enabled. The Neural Network consisted of two hidden layers with 64 and 32 neurons, respectively, with early stopping to prevent overfitting. The Voting Classifier combined all five base models (Logistic Regression, Random Forest, Gradient Boosting, SVM, and Neural Network) using soft voting.

### 2.10. Hyperparameter Tuning

Hyperparameter optimization was conducted using five-fold cross-validated grid search (GridSearchCV) on the training set, with AUC as the scoring metric. For Random Forest, the parameters explored included n_estimators (200, 300), max_depth (None, 10, 20), min_samples_split [3,9], and min_samples_leaf [2,3]. For Gradient Boosting, the grid included n_estimators (100, 200, 300), learning_rate (0.01, 0.05, 0.1), max_depth [4,9,11], and subsample (0.8, 1.0). For SVM, the tuning grid covered C (0.1, 1, 10), kernel (radial basis function), and gamma (scale, auto). The full hyperparameter search grid and best-selected values are reported in Supplementary Table S5.

### 2.11. Model Evaluation

The dataset was split into training (70%) and testing (30%) sets using stratified random sampling to preserve outcome proportions. Model performance was evaluated on the held-out test set using the following metrics: area under the receiver operating characteristic curve (AUC), accuracy, precision, recall, and F1-score. Five-fold stratified cross-validation was performed on the training set to assess model stability. The same five stratified folds (random_state = 42) were used across all algorithms and both feature tiers to ensure direct comparability of cross-validation AUC scores. Feature importance was derived from the Random Forest Gini impurity-based importance scores, while logistic regression coefficients provided directional interpretability of predictor effects.

### 2.12. Software and Reproducibility

All analyses were performed using Python 3.9.6 with scikit-learn (version 1.6.1) for machine learning, imbalanced-learn (version 0.12.4) for SMOTE, SHAP (version 0.49.1) for model interpretability, SciPy (version 1.13.1) for statistical testing, and matplotlib for visualization. Statistical comparisons of baseline characteristics between groups used the Mann–Whitney U test for continuous variables and chi-squared or Fisher’s exact test for categorical variables. A two-sided *p*-value of less than 0.05 was considered statistically significant. Given the large number of baseline comparisons (>40 variables across two outcome groups), the risk of type I error inflation is acknowledged. No formal multiple-comparison correction (e.g., Bonferroni or FDR) was applied to the descriptive baseline comparisons, as these were exploratory and intended to characterize the cohort rather than test specific hypotheses. Borderline-significant *p*-values (0.01 < *p* < 0.05) should be interpreted with appropriate caution (>40 variables across two outcome groups), and the risk of type I error inflation is acknowledged. No formal multiple-comparison correction (e.g., Bonferroni or FDR) was applied to the descriptive baseline comparisons, as these were exploratory and intended to characterize the cohort rather than test specific hypotheses. Borderline-significant *p*-values (0.01 < *p* < 0.05) should be interpreted with appropriate caution.

## 3. Results

### 3.1. Study Population

**Figure 1:**
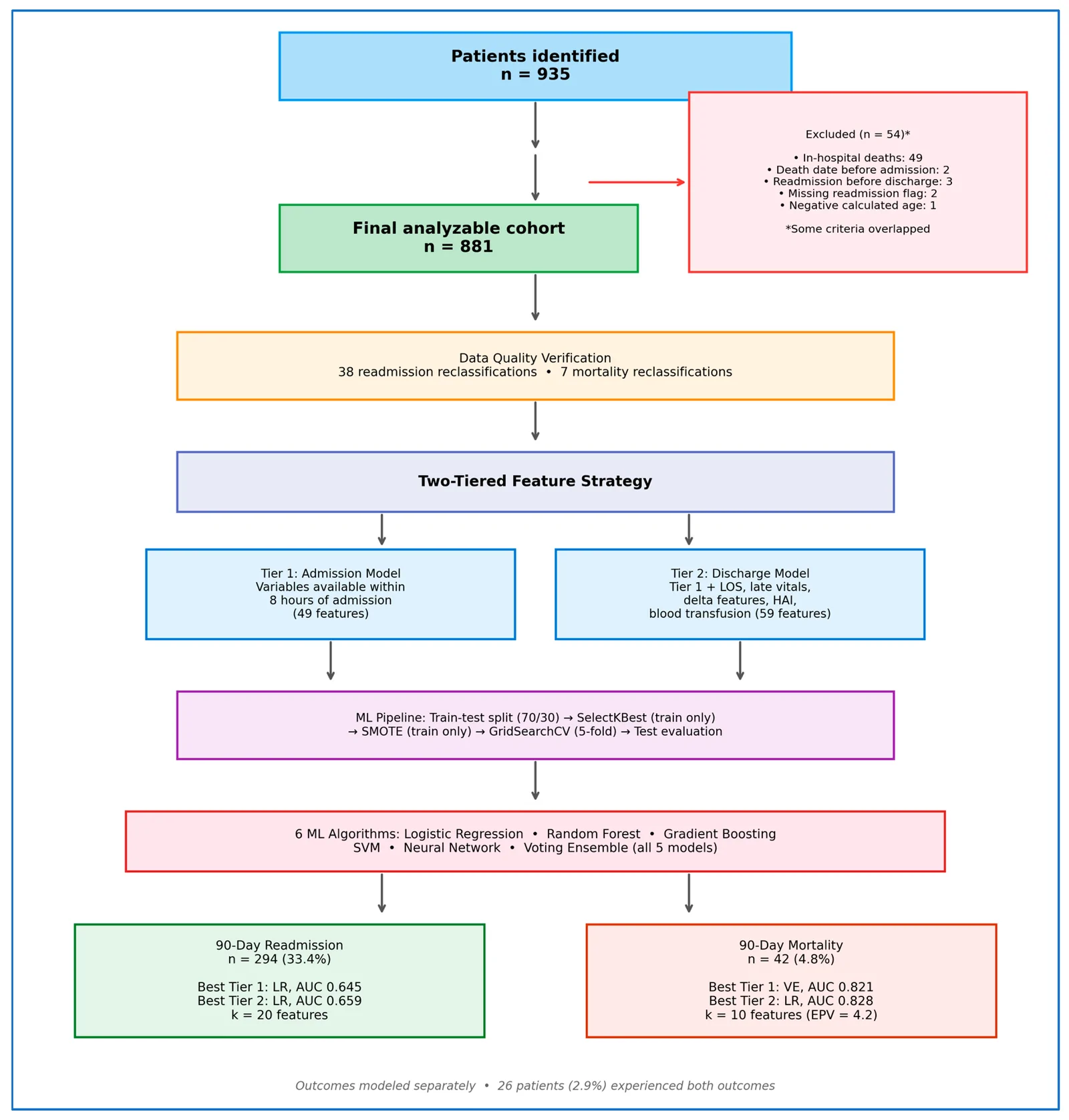
Patient selection flow diagram. From 935 identified patients, 54 were excluded, leaving 881 for analysis. Outcomes were 90-day readmission (*n* = 294, 33.4%) and 90-day post-discharge mortality (*n* = 42, 4.8%), modelled using admission-only and admission-plus-discharge features.

Of 935 patients in the original dataset, 53 were excluded, yielding a final analyzable cohort of 881 patients (Figure 1). Exclusions comprised 49 date-verified in-hospital deaths, two patients with erroneous death dates preceding admission, three patients with readmission dates preceding discharge, two patients with missing readmission flags, and one patient with a negative calculated age. Some exclusion criteria overlapped.

Among the 881 analyzable patients, 294 (33.4%) were readmitted within 90 days of discharge, and 42 (4.8%) died within 90 days of discharge. Twenty-six patients (2.9%) experienced both outcomes. The two outcomes were modelled separately.

### 3.2. Baseline Characteristics

#### 3.2.1. Readmission Cohort

Patients who were readmitted within 90 days were older, though this difference did not reach statistical significance (median 63.8 vs. 60.1 years, *p* = 0.085), had longer hospital stays (median 5.0 vs. 4.0 days, *p* = 0.002), and were more likely to carry a do-not-resuscitate (DNR) code status (13.4% vs. 5.9%, *p* < 0.001) compared with those not readmitted (Table 1). Readmitted patients had significantly greater prior healthcare utilization, including more admissions in the preceding 3 months (*p* < 0.001), admissions in the preceding 12 months (*p* < 0.001), and emergency department presentations in the preceding 12 months (*p* < 0.001). Among comorbidities, heart failure (25.0% vs. 16.6%, *p* = 0.004), chronic obstructive pulmonary disease (22.3% vs. 11.7%, *p* < 0.001), acute kidney injury (25.3% vs. 18.6%, *p* = 0.027), and chronic kidney disease (22.9% vs. 16.9%, *p* = 0.041) were significantly more prevalent in the readmitted group. Readmitted patients also had lower hemoglobin (median 10.7 vs. 11.6 g/dL, *p* < 0.001), lower albumin levels (median 37.0 vs. 39.0 g/L, *p* < 0.015), and higher alkaline phosphatase (median 90.0 vs. 86.0 U/L, *p* = 0.050).

#### 3.2.2. Mortality Cohort

Patients who died within 90 days of discharge were markedly older (median 75.1 vs. 60.5 years, *p* < 0.001), had substantially longer hospital stays (median 9.0 vs. 5.0 days, *p* < 0.001), and demonstrated strikingly different functional profiles, with 51.2% being fully dependent compared with 18.1% of survivors (*p* < 0.001) (Table 2). DNR status was present in 44.2% of patients who died versus 6.6% of survivors (*p* < 0.001). The burden of comorbidities was markedly higher in the mortality group: 83.7% had three or more comorbidities (vs. 45.5%, *p* < 0.001), with significantly higher prevalence of diabetes mellitus (69.8% vs. 43.6%, *p* = 0.001), hypertension (67.4% vs. 43.5%, *p* = 0.003), cardiovascular disease (51.2% vs. 30.2%, *p* = 0.006), heart failure (34.1% vs. 18.4%, *p* = 0.001), and cerebrovascular disease (48.8% vs. 18.4%, *p* < 0.001). Hospital-acquired infection was present in 32.6% of deaths versus 4.1% of survivors (*p* < 0.001). Laboratory findings revealed significantly lower hemoglobin (9.5 vs. 11.4 g/dL, *p* < 0.001), lower albumin (28.0 vs. 39.0 g/L, *p* < 0.001), higher C-reactive protein (34.1 vs. 16.0 mg/L, *p* < 0.001), higher white blood cell count (8.5 vs. 7.0 × 10^9^/L, *p* = 0.003), and higher urea (8.1 vs. 4.8 mg/dL, *p* < 0.001) in the mortality group.

### 3.3. Feature Selection

For the readmission admission model, the top 20 selected features, ranked by ANOVA F-score, were emergency department presentations in the last 12 months (F = 27.2), admissions in the last 12 months (F = 24.2), code status (F = 22.0), admissions in the preceding 3 months (F = 18.9), COPD/interstitial lung disease (ILD) (F = 18.3), heart failure (F = 15.6), calcium (F = 14.6), albumin (F = 13.3), use of diuretics (F = 12.9), hemoglobin (F = 10.9), alkaline phosphatase (F = 10.5), comorbidities (F = 10.2), number of regular medications (F = 9.0), use of anticoagulants (F = 8.7), blood transfusion (F = 8.2), acute kidney injury (F = 6.8), chronic kidney disease (F = 6.2), need for oxygen (F = 5.6), age (F = 5.5), and urea (F = 5.1).

For the mortality admission model, the top 10 selected features were code status (F = 108.4), albumin level (F = 68.0), oxygen amount (F = 52.5), age (F = 30.4), functional status (F = 29.8), need for oxygen (F = 24.7), cerebrovascular disease (F = 23.5), comorbidities (F = 22.8), emergency department presentations (F = 20.6), and heart failure (F = 20.6). Of note, the F-scores for mortality features were substantially higher than those for readmission features, indicating stronger univariate associations between individual predictors and post-discharge death. None of the ten ICD-10-based admission diagnosis categories (respiratory, cardiovascular, cerebrovascular, infectious, gastrointestinal, metabolic, hematological, viral, renal, other) were selected by SelectKBest for either outcome, suggesting that in this general medicine population, specific admission diagnoses were less predictive than biochemical and utilization variables.

### 3.4. Model Performance: 90-Day Readmission

The readmission prediction models demonstrated modest discriminatory ability across all algorithms (Table 3). In the admission model (Tier 1), the Voting Ensemble classifier achieved the highest AUC of 0.539 (accuracy 58.5%, F1-score 33.7%). Logistic Regression achieved an AUC of 0.645 with recall of 51.1% among Tier 1 models. Random Forest, Gradient Boosting, and SVM achieved AUCs of 0.560, 0.550, and 0.560, respectively. After hyperparameter tuning, the Random Forest model did not meaningfully improve (AUC 0.617), nor did the Gradient Boosting model (AUC 0.618).

**Figure 2:**
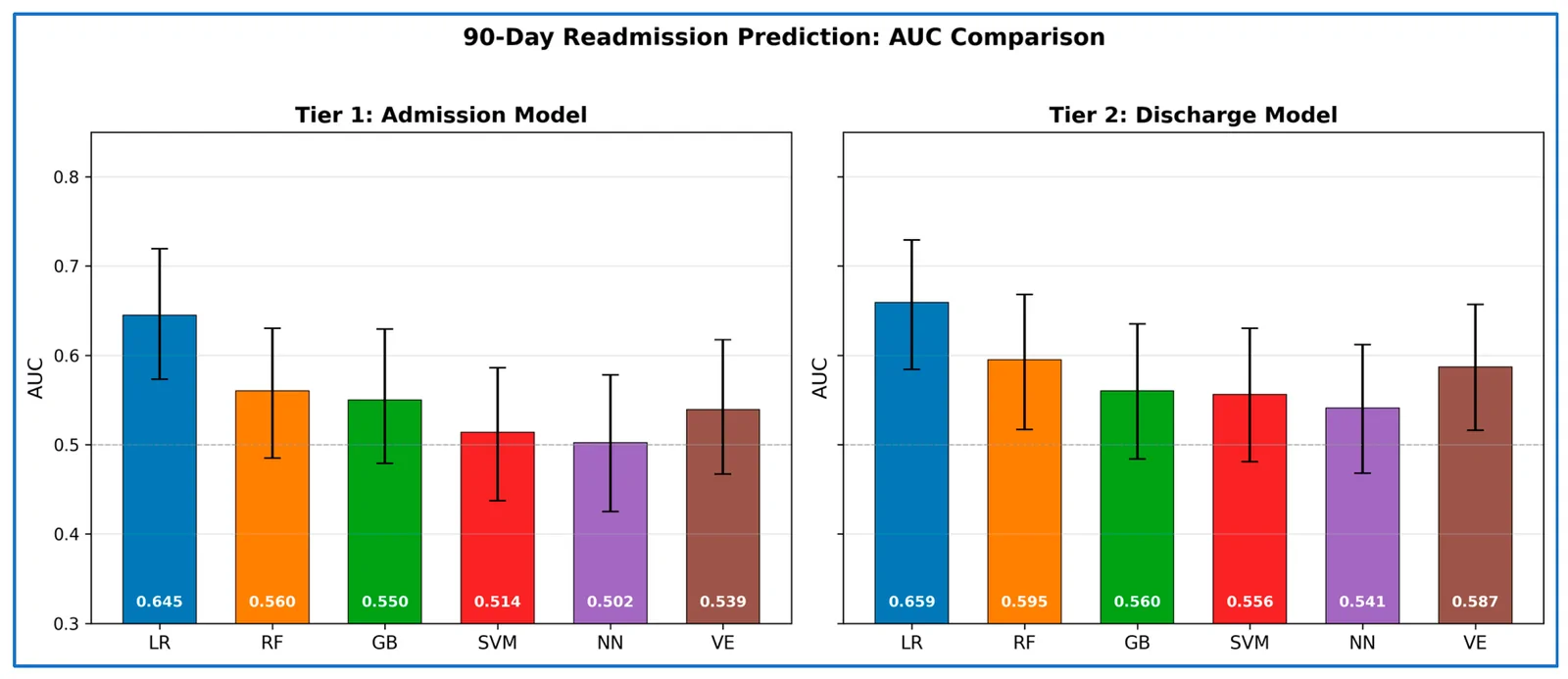
Comparative bar chart of area under the receiver operating characteristic curve (AUC) for 90-day readmission prediction across six machine learning classifiers. **Left panel**: admission model (Tier 1); **right panel**: discharge model (Tier 2). Abbreviations: AUC, area under the curve; SVM, support vector machine.

The discharge model (Tier 2), which incorporated length of stay, late vital signs, delta features, and hospital-acquired infection, demonstrated nearly identical performance to the admission model (Figure 2). The Voting Ensemble again achieved the highest AUC at 0.587 (accuracy 65.3%, F1-score 40.3%). The modest improvement from Tier 1 to Tier 2 (change in AUC of 0.014 for Logistic Regression) suggests that post-discharge readmission risk is predominantly determined by patient-level characteristics known at admission rather than by factors accruing during hospitalization.

### 3.5. Model Performance: 90-Day Mortality

**Figure 3:**
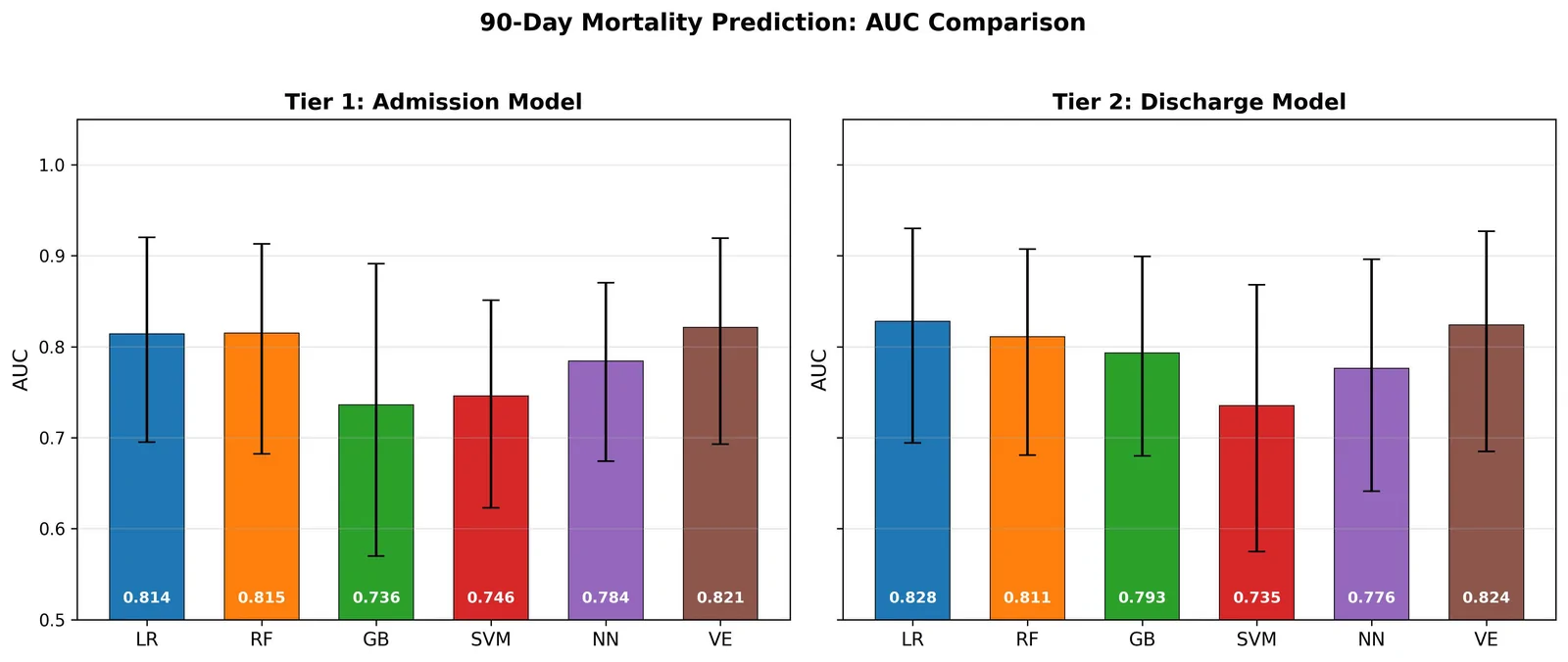
Comparative bar chart of area under the receiver operating characteristic curve (AUC) for 90-day mortality prediction across six machine learning classifiers. **Left panel**: admission model (Tier 1); **right panel**: discharge model (Tier 2). The Voting Ensemble achieved the highest Tier 1 AUC of 0.821 (Tier 1) and 0.776 (Tier 2). Error bars represent bootstrap 95% confidence intervals (1000 iterations).

The mortality prediction models achieved substantially higher discriminatory performance than the readmission models (Table 4, Figure 3). In the admission model (Tier 1), the Voting Ensemble achieved the highest AUC of 0.784 (accuracy 91.7%, precision 33.3%). Logistic Regression achieved an AUC of 0.814 with the highest recall (46.2%). The Random Forest model achieved an AUC of 0.824, while the Voting Ensemble reached 0.821. All models demonstrated high cross-validation AUC scores during training (ranging from 0.930 to 0.996). However, the drop from cross-validation to test performance was more pronounced for tree-based models (Random Forest, Gradient Boosting), suggesting moderate overfitting despite SMOTE balancing. Brier scores ranged from 0.056 (Random Forest) to 0.122 (Logistic Regression). Calibration slopes exceeded 1.0 for most models (Supplementary Table S2, Figure S1).

### 3.6. Feature Importance and Interpretability

**Figure 4:**
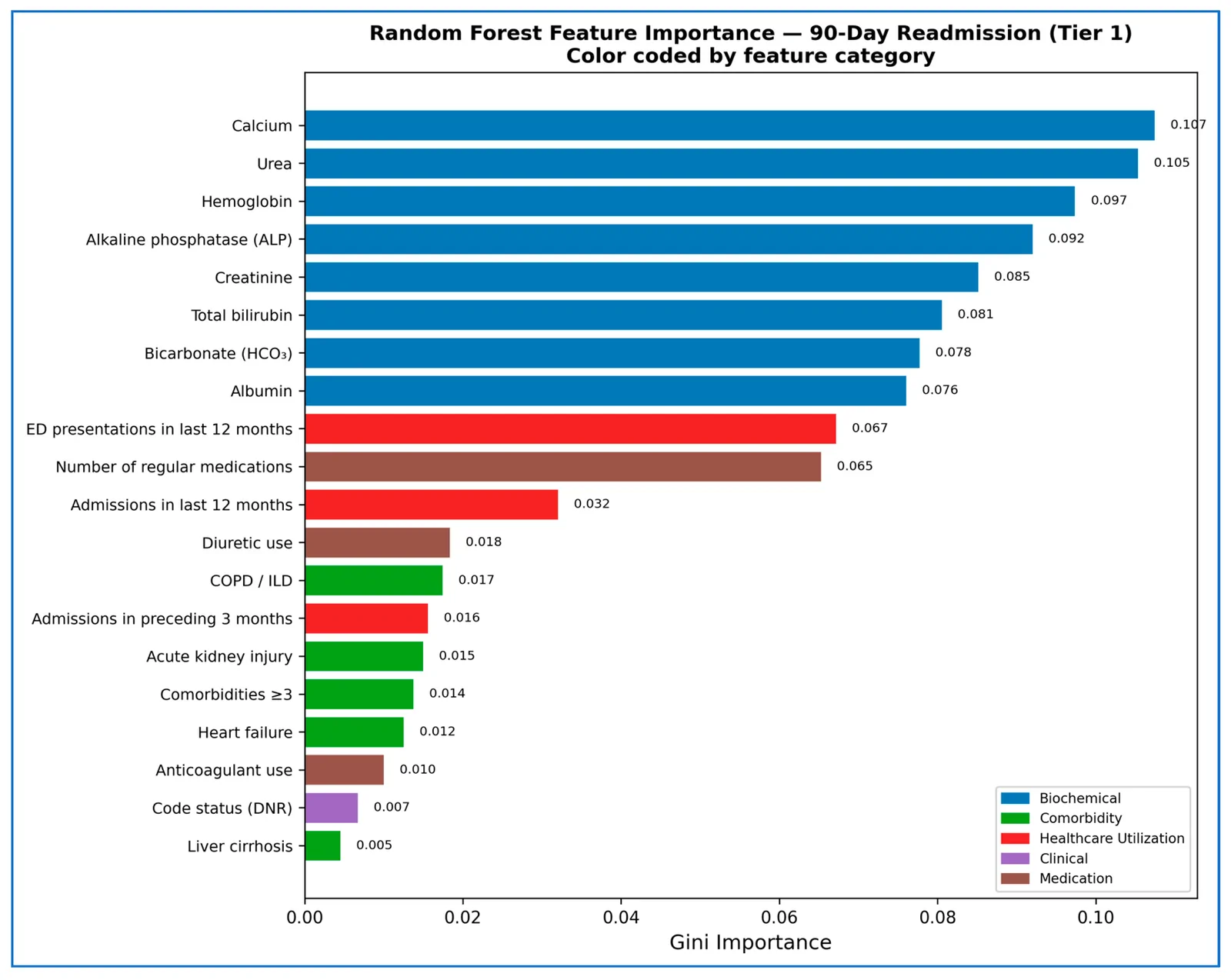
Random Forest feature importance (Gini impurity) for 90-day readmission prediction, admission model. Biochemical markers dominated the top predictors, accounting for 67.0% of total importance. Bars are colour-coded by feature category (see legend).

For the readmission admission model, Random Forest feature importance analysis (Gini impurity) identified biochemical markers as the dominant predictors (Figure 4). Alkaline phosphatase ranked first (importance = 0.128), followed by hemoglobin (0.117), urea (0.107), calcium (0.106), age (0.105), and albumin (0.092). Biochemical markers alone accounted for 67.0% of total importance; including age (demographic), the top six features represented 73.9% of total importance. Healthcare utilization features—ED presentations (0.076), number of medications (0.063), and prior admissions (0.043 and 0.025)—contributed 20.7%. Individual comorbidities each contributed modestly (all <0.025), suggesting that no single diagnosis dominates readmission prediction in general medicine.

For the mortality admission model, the feature importance profile differed markedly. Age (0.261) and albumin (0.252) together accounted for 51.4% of total importance, followed by ED presentations (0.151) and code status (0.088). Unlike readmission, where importance was distributed across many features, mortality prediction was concentrated in two dominant predictors.

**Figure 5:**
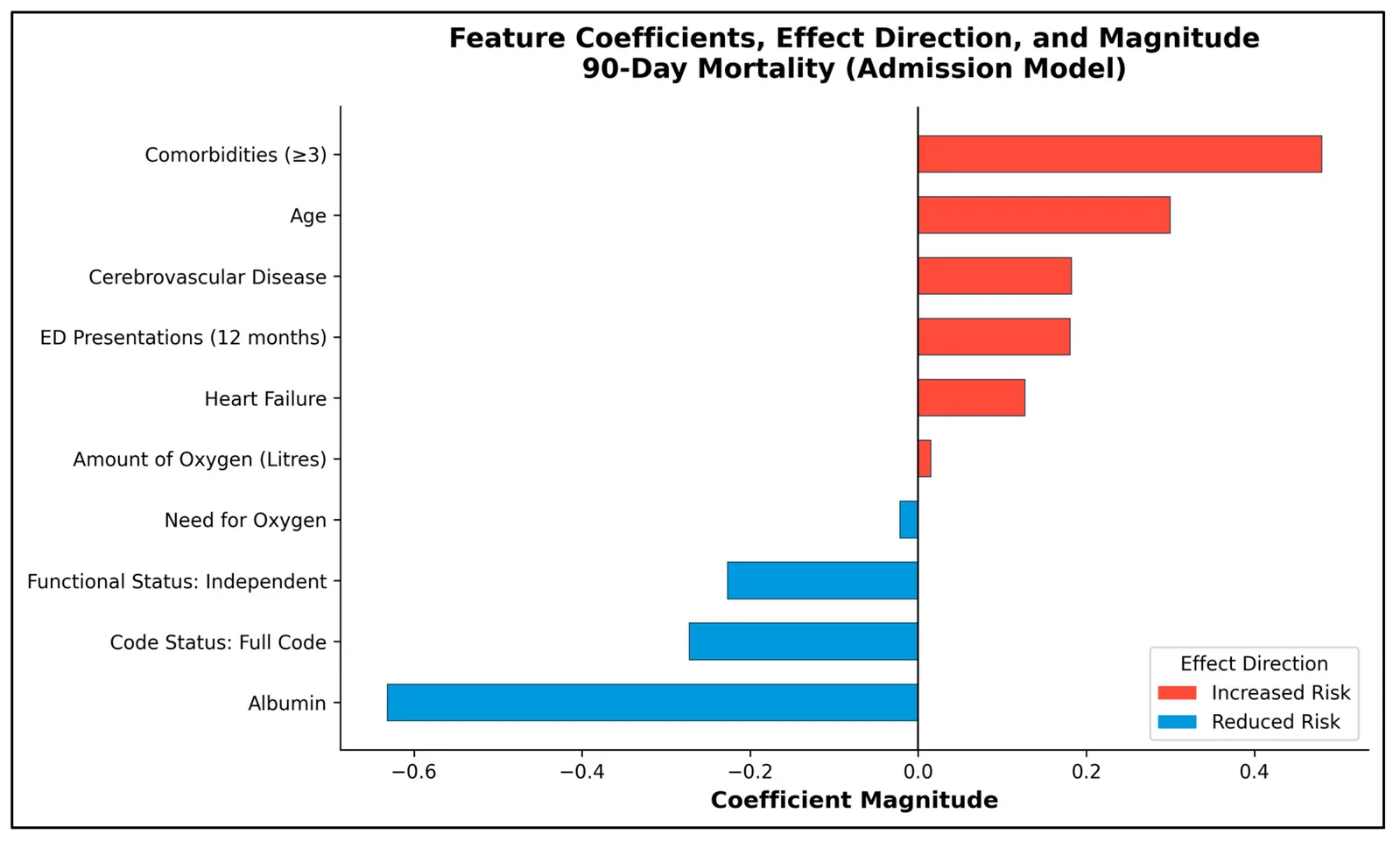
Logistic regression standardized coefficients for 90-day mortality prediction, admission model. Red indicates increased risk; blue indicates protective associations.

Logistic regression coefficients revealed the direction of these associations (Figure 5). Comorbidities ≥ 3 (coefficient = 0.480), age (0.300), cerebrovascular disease (0.183), and ED presentations (0.181) were the strongest risk factors. Albumin (−0.595), full code status (−0.272), and functional independence (−0.227) were protective. The strong protective effect of albumin is consistent with its established role as a marker of nutritional status and disease severity (Figure 5). SHAP analysis provided patient-level interpretability. For readmission, top features by mean |SHAP| were calcium (0.140), blood transfusion (0.103), and COPD/ILD (0.095). For mortality, top features were comorbidities ≥ 3 (1.080), albumin (0.542), and ED presentations (0.456) (Supplementary Figures S3 and S4).

**Figure 6:**
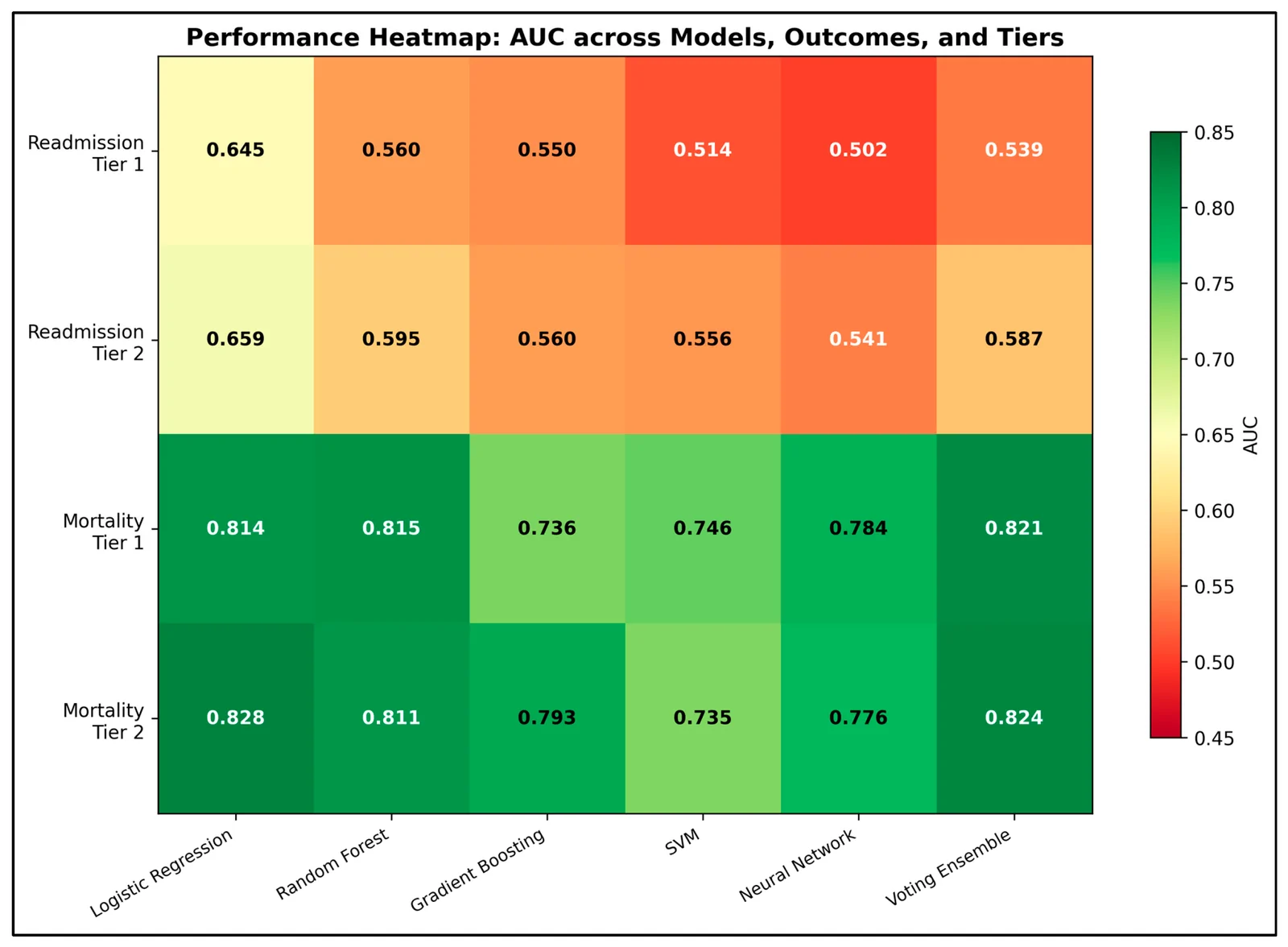
Performance heatmap summarizing AUC values across all 28 model–outcome–tier combinations (7 classifiers × 2 outcomes × 2 tiers). Colour coding: Green indicates higher discriminatory performance; red indicates lower performance. All readmission models clustered in a narrow AUC range of 0.502–0.659, while mortality models ranged from 0.735 to 0.828, demonstrating the substantially greater predictability of 90-day post-discharge mortality compared with readmission. The Voting Ensemble and Neural Network consistently ranked among the top performers for readmission and mortality, respectively.

Figure 6 presents a heatmap summarizing AUC performance across all model-outcome-tier combinations. The most notable finding is the striking disparity between readmission and mortality prediction: all readmission models clustered between AUC 0.659 and 0.659, while mortality models ranged from 0.735 to 0.828. Within each outcome, the minimal difference between admission (Tier 1) and discharge (Tier 2) models suggests that the addition of inpatient trajectory features provided limited incremental predictive value. Logistic Regression consistently ranked among the top performers for both mortality and readmission prediction.

## 4. Discussion

This retrospective cohort study of 881 general medicine inpatients provides a comprehensive characterization of the clinical, functional and biochemical determinants of 90-day hospital readmission and mortality. Our findings demonstrate that both outcomes are driven by overlapping but distinct risk profiles, with mortality being associated with a markedly higher burden of multimorbidity, functional dependence and systemic inflammation, while readmission was more closely linked to prior healthcare utilization, cardiorespiratory disease and renal dysfunction. These results confirm earlier evidence and expand current understanding of post-discharge outcomes in general medicine settings.

### 4.1. 90-Day Hospital Readmission

The 90-day readmission rate in our cohort is broadly consistent with published data from general medicine settings, where unplanned readmission rates within 90 days have been reported in the range of 20–35% [5,31]. Readmitted patients were older, though this difference did not reach statistical significance (median 63.8 vs. 60.1 years) and had longer index hospital stays (median 5.0 vs. 4.0 days). Prolonged length of stay during the index admission has been consistently identified as a readmission risk factor, possibly reflecting greater illness severity or incomplete clinical resolution at the time of discharge [5].

One of the strongest predictors of readmission in our study was prior healthcare utilization, including hospitalizations within the past 3 and 12 months, as well as emergency department visits during the previous 12 months. This is consistent with the large body of literature identifying prior hospitalization as the single strongest predictor of future readmission [5,32]. These patients represent a high-utilization subgroup who are likely to benefit from targeted post-discharge interventions that include transitional care programmes [33], enhanced community support and proactive outpatient follow-up.

Among comorbidities, heart failure and COPD were the most strongly associated with readmission, with prevalence rates of 25.0% and 22.3% respectively in the readmitted group, compared with 16.6% and 11.7% in non-readmitted patients. These diagnoses are the leading causes of unplanned readmission in most healthcare systems and are the focus of major quality improvement initiatives internationally [34,35,36]. Both conditions are characterized by episodic decompensation, and their management requires careful discharge planning, patient education and coordinated community follow-up. The high readmission burden associated with these diagnoses in our cohort highlights the need for disease-specific discharge protocols and early post-discharge review.

Acute kidney injury (AKI) and chronic kidney disease (CKD) were also significantly more prevalent in the readmitted group (25.3% vs. 18.6% and 22.9% vs. 16.9%, respectively). Renal impairment is increasingly recognized as a key driver of hospital readmission, both as an independent contributor and through its interaction with cardiovascular disease, fluid dysregulation and medication complexity [37,38]. The co-occurrence of heart failure and renal disease in readmitted patients in our cohort is consistent with the cardiorenal syndrome pattern, in which bidirectional dysfunction between the heart and kidneys amplifies the risk of clinical deterioration and readmission [39].

Readmitted patients had significantly lower hemoglobin (10.7 vs. 11.6 g/dL) and albumin levels (37.0 vs. 39.0 g/L), as well as higher alkaline phosphatase (90.0 vs. 86.0 U/L). Anemia at discharge has been associated with increased readmission risk in multiple studies, potentially reflecting unresolved acute illness, nutritional deficiency, or underlying chronic disease [40]. The modest but statistically significant difference in albumin, while not as extreme as in the mortality group, may reflect subclinical malnutrition or low-grade inflammation. Elevated alkaline phosphatase may suggest hepatobiliary or bone pathology, but it is also associated with worse outcomes across cardiovascular, renal, neurological, and critical illness settings [41,42]. Together, these laboratory differences may identify patients with greater systemic vulnerability and higher risk of rehospitalization, supporting the value of closer discharge planning, follow-up, and targeted clinical review.

### 4.2. 90-Day Mortality

Patients who died within 90 days were substantially older (median 75.1 vs. 60.5 years, *p* < 0.001), a finding that is well established in the literature [43,44]. Age is a recognized independent predictor of post-discharge mortality, which reflects the cumulative physiological decline, reduced physiological reserve and greater prevalence of life-limiting comorbidities in older patients [43,45,46]. The 15-year difference in median age between those who died and survivors indicates that age should be given special consideration in general medicine settings when using targeted mortality risk stratification techniques.

A particularly remarkable finding was that 51.2% of patients who died were fully functionally dependent, compared with only 18.1% of survivors (*p* < 0.001). Functional dependency is a well-validated prognostic marker in hospitalized older adults and has been incorporated into several risk stratification [47,48,49] tools, including the Hospital Admission Risk Profile (HARP) and the Identification of Seniors at Risk (ISAR) score [47,48]. Our results reinforce the critical importance of formal functional assessment at the time of hospital admission as a routine component of mortality risk stratification [47,48,49].

DNR status was present in 44.2% of patients who died versus only 6.6% of survivors (*p* < 0.001), representing one of the most statistically and clinically significant differences between groups. This finding requires careful interpretation [50,51], as DNR status may reflect the severity of a patient’s underlying condition while also influencing outcomes through intentional limits on life-sustaining treatment. In serious medical illness, DNR and related treatment restriction orders have been consistently identified among the strongest predictors of short- and medium-term mortality, a relationship demonstrated across a range of clinical settings internationally [50,51] . The high prevalence of DNR status in the mortality cohort also highlights the importance of advance care planning and goals-of-care discussions during the index admission for patients identified as high risk. A sensitivity analysis excluding code status from the mortality model showed minimal change in discrimination (Logistic Regression: 0.823 vs. 0.828 with DNR, delta −0.005), confirming that mortality prediction is driven by the constellation of clinical variables underlying DNR decisions (albumin, functional status, comorbidity burden, age) rather than the DNR designation itself (Supplementary Table S6). −0.004; Logistic Regression: 0.823 vs. 0.828 with DNR).

The comorbidity burden in the mortality group was substantial with 83.7% carrying three or more comorbidities compared with 45.5% of survivors (*p* < 0.001). Specific conditions significantly associated with death included diabetes mellitus (69.8% vs. 43.6%), hypertension (67.4% vs. 43.5%), cardiovascular disease (51.2% vs. 30.2%), heart failure (39.5% vs. 18.4%) and cerebrovascular disease (48.8% vs. 18.4%). This pattern is consistent with the widely described synergistic effect of cardiometabolic multimorbidity on post-discharge survival [52,53].

Hospital-acquired infection was present in 32.6% of patients who died compared with only 4.1% of survivors (*p* < 0.001), representing one of the most clinically striking differences between groups. This finding highlights the critical importance of infection prevention strategies in hospitalized patients, as healthcare-associated infections are associated not only with prolonged length of stay but also with significantly increased short- and medium-term mortality [54]. The disproportionate burden of hospital-acquired infection among those who subsequently died may reflect both the severity of the underlying illness that predisposed patients to infection and the independent lethal contribution of the infection itself.

From a biochemical perspective, patients who died demonstrated significantly lower hemoglobin (9.5 vs. 11.4 g/dL) and albumin (28.0 vs. 39.0 g/L), alongside elevated inflammatory markers including C-reactive protein (39.5 vs. 16.0 mg/L), white blood cell count (8.4 vs. 7.0 × 10^9^/L), and urea (8.1 vs. 4.8 mmol/L). Hypoalbuminemia is a well-established marker of malnutrition, inflammation, and hepatic synthetic dysfunction, and has been consistently associated with adverse outcomes in hospitalized patients [55,56]. The elevated C-reactive protein (CRP) and leukocytosis suggest a state of systemic inflammation, which may reflect uncontrolled infection, advanced malignancy, or decompensated chronic disease. Elevated urea, reflecting impaired renal function or increased protein catabolism, neurohormonal activation and volume status, is a recognized predictor of mortality in general medical patients and forms part of validated prognostic scores such as APACHE/SAPS scores [57]. Taken together, these laboratory abnormalities represent a pattern of physiological frailty and systemic dysfunction that is highly predictive of post-discharge mortality [55,56,57,58].

### 4.3. Machine Learning Model Performance

A central finding of this study is the substantial disparity between mortality and readmission prediction performance. The best mortality model (Voting Ensemble, admission-model AUC 0.821; Logistic Regression, discharge-model AUC 0.828) achieved good discrimination, while the best readmission model (Logistic Regression, AUC 0.645) demonstrated only modest discrimination. This pattern is consistent with the broader literature, where readmission prediction has proven consistently more challenging than mortality prediction across diverse clinical settings and methodological approaches [22,23,24,56].

The modest readmission AUC of 0.645 should be interpreted in the context of the global literature on readmission prediction. Systematic reviews have consistently reported AUC values in the range of 0.60–0.70 for readmission models, regardless of the algorithm employed [25,26]. Established clinical scoring systems such as the LACE (Length of stay, Acuity, Comorbidities, Emergency department visits) index [59] and the HOSPITAL (Hemoglobin, Oncology, Sodium, Procedure, Index Type, Admissions, Length of stay) score [60] typically achieve AUCs of 0.60–0.68 in external validation studies. A direct comparison was not feasible as our dataset did not capture all required component variables. The fundamental challenge of readmission prediction lies in the substantial contribution of unmeasured factors, including social determinants of health, patient health literacy and self-management capacity, medication adherence, caregiver availability, and healthcare system fragmentation, none of which are captured in routine inpatient EHR data. A recent systematic review found that machine learning algorithms did not consistently outperform logistic regression for clinical prediction tasks [56], suggesting that the performance ceiling for readmission prediction may be driven by data limitations rather tha algorithmic sophistication.

Perhaps the most operationally important finding is that discharge models (Tier 2) did not meaningfully improve upon admission models (Tier 1) for either outcome. For readmission, the AUC difference between tiers was less than 0.001, and for mortality, the best discharge model (Logistic Regression, AUC 0.828) was marginally higher than the best admission model (Voting Ensemble, AUC 0.821). This suggests that the clinical trajectory during hospitalization, as captured by delta vital signs, late vital signs, and length of stay, added minimal incremental predictive value beyond patient-level characteristics known at admission. From a clinical standpoint, this is an encouraging finding: risk stratification can be initiated on Day 1 of admission, enabling earlier mobilization of transitional care resources and discharge planning for high-risk patients.

Several methodological considerations warrant careful interpretation of the mortality prediction results. The mortality outcome had only 42 events in the full cohort (13 events in the test set), yielding an events-per-variable (EPV) ratio of 4.2 for the 10-feature mortality models. This is substantially below the commonly recommended minimum of 10–20 EPV for reliable prediction model development [30]. While the high cross-validation AUCs (0.937–0.996) suggest strong discrimination on training data, the drop to test AUCs of 0.735–0.828 indicates overfitting, particularly for tree-based models. The application of SMOTE to address the 5% mortality event rate meant that approximately 95% of minority class training examples were synthetic, which may inflate apparent model performance and produce optimistic estimates of discrimination [61,62]. As a single-centre study from Oman, these models may not generalize to healthcare systems with different patient demographics or clinical workflows [57]. These results should therefore be considered hypothesis-generating and require validation in larger, independent cohorts before clinical deployment.

Calibration assessment revealed that while mortality models demonstrate good discrimination, predicted probabilities show some miscalibration. Brier scores ranged from 0.059 to 0.127 for mortality. Calibration slopes exceeded 1.0 for most models, indicating overconfident predictions typical of small-sample models [62] Discrimination (AUC) measures a model’s ability to rank-order patients by risk, but does not assess whether predicted probabilities accurately reflect observed event rates. Poor calibration can render a well-discriminating model clinically misleading, particularly for individual-level risk communication [62]. Future studies should report calibration plots and the Hosmer–Lemeshow statistic or calibration slopes to provide a more complete assessment of model performance. Large-scale studies using comprehensive EHR data have demonstrated that deep learning approaches can achieve strong predictive performance when sufficient sample sizes and diverse data sources are available [58,63].

A sensitivity analysis comparing SMOTE, class weighting, and no balancing confirmed that mortality discrimination was robust across approaches (best AUC: SMOTE 0.828, class weighting 0.873, no balancing 0.859; Supplementary Table S1). A reduced feature set (k = 5, EPV = 8.6) achieved AUC 0.807 (Supplementary Table S3). Temporal validation (train 2022, *n* = 479; test 2023, *n* = 402) confirmed stability: mortality LR AUC 0.891 (95% CI: 0.825–0.940), readmission LR AUC 0.673 (95% CI: 0.603–0.738; Supplementary Table S4).

### 4.4. Clinical Implications

The findings of this study carry several implications for clinical practice. First, the observation that admission-day variables alone achieved comparable discriminatory performance to discharge-augmented models suggests that clinically meaningful risk stratification can be performed on the day of hospital admission, without waiting for the clinical trajectory to unfold during the hospitalization. This has important operational implications, as it enables early identification of high-risk patients who may benefit from targeted interventions such as enhanced discharge planning, transitional care coordination, and proactive outpatient follow-up. Second, the integration of ML-derived risk scores with established clinical scoring systems such as the HOSPITAL score [60] and the LACE index [59] warrants investigation in future studies, as hybrid approaches combining data-driven predictions with validated clinical instruments may offer incremental improvements in risk stratification accuracy. Third, prospective validation in an independent cohort is an essential prerequisite before any ML-based prediction tool can be recommended for clinical deployment. The present models should be regarded as hypothesis-generating rather than ready for bedside implementation. Given the low EPV ratio (4.2 for mortality), the reliance on a single centre, and the absence of external validation, these results require replication in larger, multi-site cohorts before any clinical translation can be considered. Finally, the relatively poor performance of readmission prediction models across all algorithms reinforces the need for post-discharge data integration, including social determinants of health, medication adherence, and outpatient follow-up engagement, to meaningfully improve readmission risk stratification beyond what is achievable with inpatient clinical data alone. Decision curve analysis (Supplementary Figure S2) demonstrated net benefit over treat-all and treat-none strategies across clinically relevant thresholds (5–30%).

### 4.5. Strengths and Limitations

This study has several notable strengths. The relatively large sample size of 881 patients provides adequate statistical power to detect clinically meaningful differences in outcomes. The comprehensive characterization of baseline demographics, comorbidities, functional status, laboratory parameters and prior healthcare utilization allows for a multidimensional analysis of risk factors. The use of 90-day outcomes, rather than the more commonly reported 30-day endpoints, provides a more complete picture of post-discharge morbidity and mortality.

However, several limitations must be acknowledged. As a retrospective single-centre study, our findings may not be fully generalizable to other institutions or healthcare systems with different patient populations, resource availability, or care practices. Retrospective data collection is subject to documentation bias and may result in incomplete capture of all relevant clinical variables. We were unable to account for post-discharge factors such as medication adherence, social support and access to primary care, which are known to influence both readmission and mortality. The exclusion of clinically relevant biomarkers with high missingness rates (BNP: 82%, ferritin: 82%, lactate: 33%, troponin: 37%) may have limited model performance, particularly for heart failure-related readmissions. Future studies with more complete laboratory panels may achieve higher discrimination. Additionally, as we report association data from univariate analyses of baseline characteristics, the independent contribution of each variable to outcomes in a multivariate model warrants further investigation through multivariable logistic regression analysis.

## 5. Conclusions

In this retrospective cohort of general medicine patients, 90-day mortality was predominantly driven by advanced age, functional dependence, multimorbidity, hospital-acquired infection and a biochemical profile reflecting systemic inflammation, malnutrition and organ dysfunction. In contrast, 90-day readmission was more strongly associated with prior healthcare utilization, cardiorespiratory and renal comorbidities and anemia. These findings identify actionable targets for quality improvement initiatives aimed at reducing post-discharge morbidity in general medicine populations. These findings should be considered hypothesis-generating given the low events-per-variable ratio, single-centre design, and absence of external validation. Future prospective research incorporating external validation in independent cohorts, multivariable regression analysis, calibration assessment, and post-discharge follow-up data is necessary before clinical deployment of risk prediction models in this population.

## Figures and Tables

**Table 1:** Baseline demographic, clinical, and biochemical characteristics of the study cohort stratified by 90-day readmission status (*N* = 881).

Variable	Total (*n* = 881)	Readmitted (*n* = 292)	Not Readmitted (*n* = 590)	*p*-Value
Age, years, median (IQR)	61.3 (42.6–74.3)	63.8 (42.7–76.6)	60.1 (42.3–73.4)	0.085
Female sex, *n* (%)	450 (51.0%)	151 (51.7%)	299 (50.7%)	0.828
Length of stay, days, median (IQR)	5.0 (3.0–8.0)	5.0 (3.0–9.0)	4.0 (3.0–8.0)	0.002
Healthcare utilization				
Admissions in preceding 3 months	0.0 (0.0–0.0)	0.0 (0.0–0.0)	0.0 (0.0–0.0)	<0.001
Admissions in last 12 months	0.0 (0.0–1.0)	0.0 (0.0–1.0)	0.0 (0.0–0.0)	<0.001
ED presentations in last 12 months	1.0 (0.0–2.0)	1.0 (0.0–3.0)	0.0 (0.0–2.0)	<0.001
Clinical characteristics				
Full functional dependence, *n* (%)	174 (19.7%)	58 (19.9%)	116 (19.7%)	1.000
DNR status, *n* (%)	74 (8.4%)	39 (13.4%)	35 (5.9%)	<0.001
Temperature within 8 h, °C	36.7 (36.5–36.8)	36.7 (36.5–36.8)	36.7 (36.5–36.8)	0.692
Heart rate within 8 h, bpm	79.0 (70.0–83.0)	79.0 (70.0–83.0)	80.0 (70.0–83.0)	0.732
Need for supplemental O_2_, *n* (%)	81 (9.2%)	34 (11.6%)	47 (8.0%)	0.098
O_2_ amount, litres	0.0 (0.0–0.0)	0.0 (0.0–0.0)	0.0 (0.0–0.0)	0.102
Smoking, *n* (%)	101 (11.5%)	34 (11.6%)	67 (11.4%)	0.989
Medications				
Number of regular medications	4.0 (2.0–7.0)	5.0 (2.0–7.2)	4.0 (1.0–7.0)	0.009
Polypharmacy (≥5 medications), *n* (%)	424 (48.1%)	151 (51.7%)	273 (46.3%)	0.147
Antiepileptic use, *n* (%)	78 (8.9%)	21 (7.2%)	57 (9.7%)	0.273
Diuretic use, *n* (%)	216 (24.5%)	89 (30.5%)	127 (21.5%)	0.005
Anticoagulant use, *n* (%)	121 (13.7%)	53 (18.2%)	68 (11.5%)	0.010
Comorbidities				
≥3 comorbidities, *n* (%)	418 (47.4%)	156 (53.4%)	262 (44.4%)	0.014
Diabetes mellitus, *n* (%)	396 (44.9%)	136 (46.6%)	260 (44.1%)	0.527
Hypertension, *n* (%)	394 (44.7%)	126 (36.6%)	268 (45.4%)	0.571
Dyslipidemia, *n* (%)	240 (27.2%)	86 (29.5%)	154 (26.1%)	0.331
Cardiovascular disease, *n* (%)	275 (31.2%)	103 (35.3%)	172 (29.2%)	0.077
Heart failure, *n* (%)	171 (19.4%)	73 (25.0%)	98 (16.6%)	0.004
COPD/ILD, *n* (%)	134 (15.2%)	65 (22.3%)	69 (11.7%)	<0.001
Cerebrovascular disease, *n* (%)	175 (19.8%)	62 (21.2%)	113 (19.2%)	0.523
Active malignancy, *n* (%)	34 (3.9%)	6 (2.1%)	28 (4.7%)	0.077
Dementia, *n* (%)	56 (6.3%)	21 (7.2%)	35 (5.9%)	0.565
AKI, *n* (%)	184 (20.9%)	74 (25.3%)	110 (18.6%)	0.027
CKD, *n* (%)	167 (18.9%)	67 (22.9%)	100 (16.9%)	0.041
Liver cirrhosis, *n* (%)	28 (3.2%)	15 (5.1%)	13 (2.2%)	0.033
Hospital-acquired infection, *n* (%)	48 (5.4%)	21 (7.2%)	27 (4.6%)	0.146
Biochemical parameters				
Hemoglobin, g/dL	11.3 (9.6–12.9)	10.7 (9.2–12.6)	11.6 (9.9–13.0)	<0.001
Albumin, g/L	38.0 (32.0–43.0)	37.0 (31.0–42.0)	39.0 (33.0–43.0)	0.015
Alkaline phosphatase, U/L	87.0 (69.0–119.0)	90.0 (71.0–129.0)	86.0 (68.0–114.0)	0.050
Calcium, mmol/L	2.30 (2.23–2.38)	2.32 (2.25–2.39)	2.30 (2.22–2.38)	0.003
C-reactive protein, mg/L	17.0 (5.0–43.0)	19.0 (6.0–36.6)	16.0 (5.0–43.0)	0.526
Creatinine, µmol/L	72.0 (55.0–100.5)	72.0 (54.0–105.5)	72.0 (56.0–99.0)	0.990
eGFR, mL/min/1.73 m^2^	89.0 (58.0–90.0)	86.0 (56.0–90.0)	90.0 (61.0–90.0)	0.294
Urea, mmol/L	4.9 (3.4–8.1)	5.2 (3.7–9.1)	4.8 (3.3–7.5)	0.044
Platelet count, ×10^9^/L	273.5 (204.8–356.2)	278.5 (215.0–363.8)	268.5 (201.0–349.8)	0.055
WBC, ×10^9^/L	7.0 (5.1–9.1)	7.0 (5.1–9.3)	7.0 (5.1–8.9)	0.476
Hematocrit, %	0.4 (0.3–0.4)	0.3 (0.3–0.4)	0.4 (0.3–0.4)	<0.001
Sodium, mmol/L	137.0 (155.7–139.0)	137.0 (155.7–139.0)	137.0 (135.0–139.0)	0.201
Potassium, mmol/L	4.2 (3.9–4.5)	4.2 (3.9–4.5)	4.2 (3.9–4.5)	0.889
HCO_3_, mmol/L	23.0 (21.0–25.0)	23.0 (21.0–25.0)	23.0 (21.0–25.0)	0.727
Chloride, mmol/L	102.0 (99.0–105.0)	102.0 (98.0–105.0)	102.0 (99.0–105.0)	0.566
ALT, U/L	19.0 (12.0–37.0)	17.0 (11.0–33.5)	20.0 (12.0–38.0)	0.151
AST, U/L	21.0 (15.0–37.0)	20.0 (15.0–35.0)	22.0 (16.0–38.0)	0.205
Bilirubin total, µmol/L	7.0 (5.0–11.0)	7.0 (5.0–11.0)	7.0 (4.0–11.0)	0.976
Glucose, mmol/L	7.6 (6.0–10.6)	7.8 (6.0–10.2)	7.6 (5.9–10.7)	0.677
Blood transfusion, *n* (%)	70 (7.9%)	35 (12.0%)	35 (5.9%)	0.003

Data are presented as median (IQR) for continuous variables and *n* (%) for categorical variables. *p*-values were calculated using Mann–Whitney U test for continuous variables and chi-square or Fisher’s exact test for categorical variables. IQR, interquartile range; COPD, chronic obstructive pulmonary disease; ILD, interstitial lung disease; AKI, acute kidney injury; CKD, chronic kidney disease; CRP, C-reactive protein; eGFR, estimated glomerular filtration rate; ALP, alkaline phosphatase; ALT, alanine aminotransferase; AST, aspartate aminotransferase; WBC, white blood cell count; HCO_3_, bicarbonate.

**Table 2:** Baseline demographic, clinical, and biochemical characteristics of the study cohort stratified by 90-day post-discharge mortality status (*N* = 881).

Variable	Total (*n* = 881)	Died (*n* = 43)	Survived (*n* = 839)	*p*-Value
Age, years, median (IQR)	61.3 (42.6–74.3)	75.1 (65.0–81.1)	60.5 (51.9–73.5)	<0.001
Female sex, *n* (%)	450 (51.0%)	27 (62.8%)	423 (50.4%)	0.154
Length of stay, days, median (IQR)	5.0 (3.0–8.0)	9.0 (6.0–16.0)	5.0 (3.0–8.0)	<0.001
Healthcare utilization				
Admissions in preceding 3 months	0.0 (0.0–0.0)	0.0 (0.0–1.0)	0.0 (0.0–0.0)	<0.001
Admissions in last 12 months	0.0 (0.0–1.0)	1.0 (0.0–2.0)	0.0 (0.0–1.0)	<0.001
ED presentations in last 12 months	1.0 (0.0–2.0)	2.0 (1.0–3.5)	0.0 (0.0–2.0)	<0.001
Clinical characteristics				
Full functional dependence, *n* (%)	174 (19.7%)	22 (51.2%)	152 (18.1%)	<0.001
DNR status, *n* (%)	74 (8.4%)	19 (44.2%)	55 (6.6%)	<0.001
Temperature within 8 h, °C	36.7 (36.5–36.8)	36.8 (36.5–37.0)	36.7 (36.5–36.8)	0.044
Heart rate within 8 h, bpm	79.0 (70.0–83.0)	82.0 (73.5–92.5)	79.0 (70.0–83.0)	0.192
Need for supplemental O_2_, *n* (%)	81 (9.2%)	11 (25.6%)	70 (8.3%)	<0.001
O_2_ amount, litres	0.0 (0.0–0.0)	0.0 (0.0–0.5)	0.0 (0.0–0.0)	<0.001
Smoking, *n* (%)	101 (11.5%)	3 (7.0%)	98 (11.7%)	0.465
Medications				
Number of regular medications	4.0 (2.0–7.0)	6.0 (4.0–8.5)	4.0 (1.0–7.0)	<0.001
Polypharmacy (≥5 medications), *n* (%)	424 (48.1%)	29 (67.4%)	395 (47.1%)	0.014
Antiepileptic use, *n* (%)	78 (8.9%)	5 (11.6%)	73 (8.7%)	0.703
Diuretic use, *n* (%)	216 (24.5%)	21 (48.8%)	195 (23.2%)	<0.001
Anticoagulant use, *n* (%)	121 (13.7%)	11 (25.6%)	110 (13.1%)	0.037
Comorbidities				
≥3 comorbidities, *n* (%)	418 (47.4%)	36 (83.7%)	382 (45.5%)	<0.001
Diabetes mellitus, *n* (%)	396 (44.9%)	30 (69.8%)	366 (43.6%)	0.001
Hypertension, *n* (%)	394 (44.7%)	29 (67.4%)	365 (43.5%)	0.003
Dyslipidemia, *n* (%)	240 (27.2%)	16 (37.2%)	224 (26.7%)	0.182
Cardiovascular disease, *n* (%)	275 (31.2%)	22 (51.2%)	253 (30.2%)	0.006
Heart failure, *n* (%)	171 (19.4%)	17 (39.5%)	154 (18.4%)	0.001
COPD/ILD, *n* (%)	134 (15.2%)	7 (16.3%)	127 (15.1%)	1.000
Cerebrovascular disease, *n* (%)	175 (19.8%)	21 (48.8%)	154 (18.4%)	<0.001
Active malignancy, *n* (%)	34 (3.9%)	4 (9.3%)	30 (3.6%)	0.078
Dementia, *n* (%)	56 (6.3%)	7 (16.3%)	49 (5.8%)	0.016
AKI, *n* (%)	184 (20.9%)	18 (41.9%)	166 (19.8%)	0.001
CKD, *n* (%)	167 (18.9%)	14 (32.6%)	153 (18.2%)	0.032
Liver cirrhosis, *n* (%)	28 (3.2%)	3 (7.0%)	25 (3.0%)	0.152
Hospital-acquired infection, *n* (%)	48 (5.4%)	14 (32.6%)	34 (4.1%)	<0.001
Biochemical parameters				
Hemoglobin, g/dL	11.3 (9.6–12.9)	9.5 (8.4–11.1)	11.4 (9.6–13.0)	<0.001
Albumin, g/L	38.0 (32.0–43.0)	28.0 (25.0–33.8)	39.0 (33.0–43.0)	<0.001
Alkaline phosphatase, U/L	87.0 (69.0–119.0)	117.0 (79.8–157.5)	86.0 (68.0–115.0)	<0.001
Calcium, mmol/L	2.3 (2.2–2.4)	2.4 (2.3–2.5)	2.3 (2.2–2.4)	<0.001
C-reactive protein, mg/L	17.0 (5.0–43.0)	39.5 (14.8–73.2)	16.0 (5.0–42.0)	<0.001
Creatinine, µmol/L	72.0 (55.0–100.5)	73.0 (42.0–96.0)	72.0 (55.0–101.0)	0.778
eGFR, mL/min/1.73 m^2^	89.0 (58.0–90.0)	85.0 (47.2–90.0)	90.0 (58.0–90.0)	0.228
Urea, mmol/L	4.9 (3.4–8.1)	8.1 (5.2–12.1)	4.8 (3.4–7.8)	<0.001
Platelet count, ×10^9^/L	273.5 (204.8–356.2)	246.0 (200.5–415.5)	275.0 (205.0–355.0)	0.791
WBC, ×10^9^/L	7.0 (5.1–9.1)	8.5 (6.7–9.9)	7.0 (5.1–9.0)	0.003
Hematocrit, %	0.4 (0.3–0.4)	0.3 (0.3–0.4)	0.4 (0.3–0.4)	<0.001
Sodium, mmol/L	137.0 (155.7–139.0)	131.8 (133.2–140.0)	137.0 (155.7–139.0)	0.968
Potassium, mmol/L	4.2 (3.9–4.5)	4.1 (3.7–4.4)	4.2 (3.9–4.5)	0.303
HCO_3_, mmol/L	23.0 (21.0–25.0)	23.0 (20.0–26.0)	23.0 (21.0–25.0)	0.410
Chloride, mmol/L	102.0 (99.0–105.0)	101.5 (98.2–105.8)	102.0 (99.0–105.0)	0.935
ALT, U/L	19.0 (12.0–37.0)	20.0 (12.0–33.0)	19.0 (12.0–37.0)	0.883
AST, U/L	21.0 (15.0–37.0)	20.5 (16.0–34.5)	21.0 (15.0–38.0)	0.959
Bilirubin total, µmol/L	7.0 (5.0–11.0)	7.0 (5.0–9.0)	7.0 (5.0–11.0)	0.551
Glucose, mmol/L	7.6 (6.0–10.6)	8.7 (8.0–10.9)	7.5 (5.9–10.6)	0.059
Blood transfusion, *n* (%)	70 (7.9%)	4 (9.3%)	66 (7.9%)	0.770

Data are presented as median (interquartile range) for continuous variables and *n* (%) for categorical variables. *p*-values were calculated using the Mann–Whitney U test for continuous variables and chi-square or Fisher’s exact test for categorical variables. Patients who died within 90 days of discharge (*n* = 43, 4.9%) were compared with survivors (*n* = 839). Abbreviations as in Table 1.

**Table 3:** Performance metrics of seven machine learning classifiers for 90-day readmission prediction: Admission model (Tier 1, upper panel) and discharge model (Tier 2, lower panel).

Model	AUC	Accuracy (%)	Precision (%)	Recall (%)	F1-Score (%)	Brier Score
Tier 1: Admission Model						
Logistic Regression	0.645	65.3	34.6	51.9	49.5	0.232
Random Forest	0.514	63.8	42.0	23.9	30.4	0.228
Gradient Boosting	0.641	65.3	46.9	39.5	39.5	0.242
SVM	0.514	54.7	33.3	36.4	34.8	0.267
Neural Network	0.502	32.4	30.9	34.1	32.4	0.398
Voting Ensemble	0.539	62.6	35.9	31.8	47.6	0.241
Tier 2: Discharge Model						
Logistic Regression	0.659	65.7	48.5	55.7	51.9	0.229
Random Forest	0.632	65.3	50.0	51.9	40.5	0.220
Gradient Boosting	0.560	64.9	35.9	35.2	40.0	0.229
SVM	0.556	60.8	39.5	34.1	36.6	0.229
Neural Network	0.541	50.0	37.8	31.8	34.6	0.359
Voting Ensemble	0.634	65.7	41.8	31.8	36.4	0.228

AUC, area under the receiver operating characteristic curve; LR, logistic regression; RF, random forest; XGB, XGBoost; SVM, support vector machine; NN, neural network; KNN, k-nearest neighbours; VE, Voting Ensemble.

**Table 4:** Performance metrics of seven machine learning classifiers for 90-day post-discharge mortality prediction.

Model	AUC	Accuracy (%)	Precision (%)	Recall (%)	F1-Score (%)	Brier Score
Tier 1: Admission Model						
Logistic Regression	0.735	82.3	17.1	46.2	25.0	0.094
Random Forest	0.679	92.8	16.7	7.7	10.5	0.071
Gradient Boosting	0.736	92.1	16.7	23.1	19.4	0.078
SVM	0.746	92.8	25.0	15.4	19.0	0.072
Neural Network	0.784	92.5	33.3	15.4	21.1	0.071
Voting Ensemble	0.830	92.5	23.1	23.1	23.1	0.068
Tier 2: Discharge Model						
Logistic Regression	0.828	83.0	17.1	46.2	25.0	0.089
Random Forest	0.811	93.2	33.3	7.7	12.5	0.062
Gradient Boosting	0.793	91.3	20.0	15.4	17.4	0.075
SVM	0.735	92.5	25.0	15.4	19.0	0.068
Neural Network	0.817	92.5	30.0	23.1	26.1	0.068
Voting Ensemble	0.815	92.8	33.3	15.4	21.1	0.063

## Data Availability

The analysis code is available at https://github.com/alalawi2/90day-readmission-ml (accessed on 24 September 2026). The clinical data are available on request from the corresponding author due to patient privacy considerations.

## Supplementary Materials

The following supporting information can be downloaded at: https://www.mdpi.com/article/10.3390/diagnostics16203337/s1.

## Abbreviations

The following abbreviations are used in this manuscript: AKI—Acute Kidney Injury; ALP—Alkaline Phosphatase; ANOVA—Analysis of Variance; AUC—Area Under the Receiver Operating Characteristic Curve; CKD—Chronic Kidney Disease; COPD—Chronic Obstructive Pulmonary Disease; CRP—C-Reactive Protein; CVD—Cardiovascular Disease; DM—Diabetes Mellitus; DNR—Do-Not-Resuscitate; ED—Emergency Department; EHR—Electronic Health Record; EPV—Events-Per-Variable; eGFR—Estimated Glomerular Filtration Rate; GIM—General Internal Medicine; HAI—Hospital-Acquired Infection; HF—Heart Failure; HOSPITAL—Hemoglobin, Oncology, Sodium, Procedure, Index Type, Admissions, Length of Stay; ICU—Intensive Care Unit; ILD—Interstitial Lung Disease; IQR—Interquartile Range; LACE—Length of Stay, Acuity, Comorbidities, Emergency Department Visits; ML—Machine Learning; MLP—Multi-Layer Perceptron; ROC—Receiver Operating Characteristic; SMOTE—Synthetic Minority Over-Sampling Technique; SVM—Support Vector Machine; TRIPOD—Transparent Reporting of a Multivariable Prediction Model for Individual Prognosis or Diagnosis.
