# Supplementary material for: Machine Learning Prediction of 90-Day Readmission and Mortality in General Internal Medicine: A Retrospective Cohort Study
Source: Diagnostics (Basel). 2026 Oct 14;16(20):3337. doi: 10.3390/diagnostics16203337 (PMC13703392; doi:10.3390/diagnostics16203337)
Supplement: Supplementary file 1 [file diagnostics-16-03337-s001.zip › diagnostics-4380765-supplementary figures.pdf]

Supplementary Figures

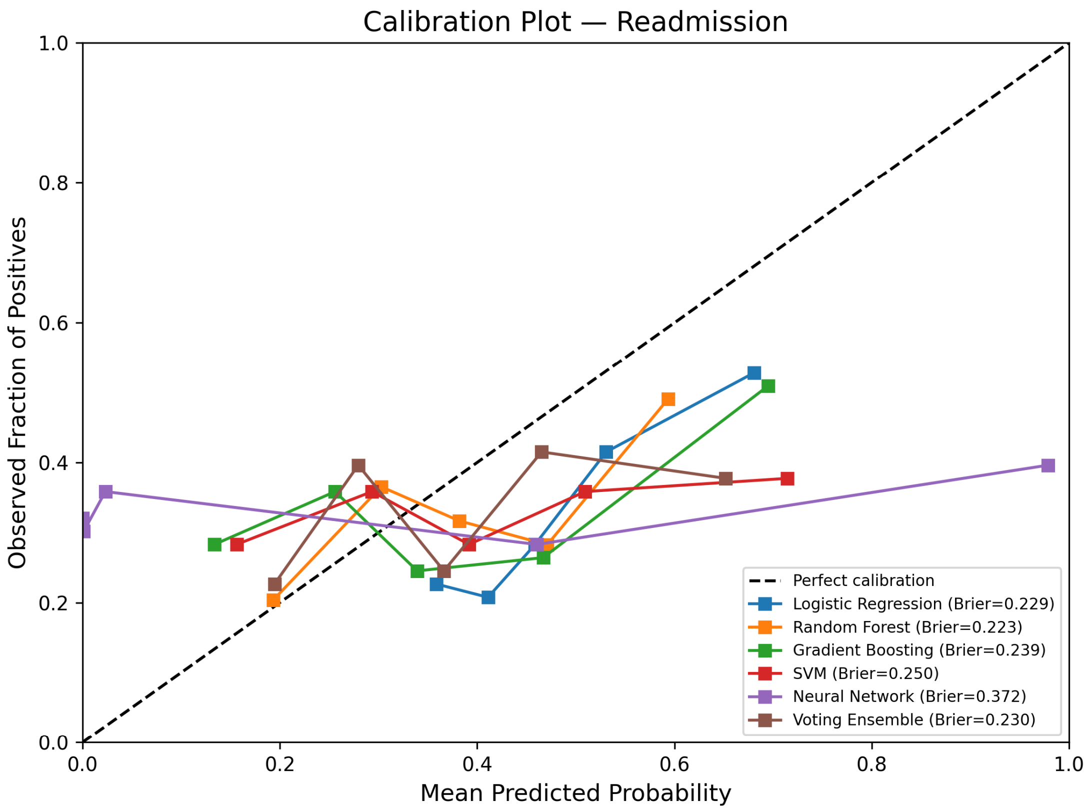
Calibration Plot — Readmission
Observed Fraction of Positives
Mean Predicted Probability
0.0
0.2
0.4
0.6
0.8
1.0
Perfect calibration
Logistic Regression (Brier=0.229)
Random Forest (Brier=0.223)
Gradient Boosting (Brier=0.239)
SVM (Brier=0.250)
Neural Network (Brier=0.372)
Voting Ensemble (Brier=0.230)

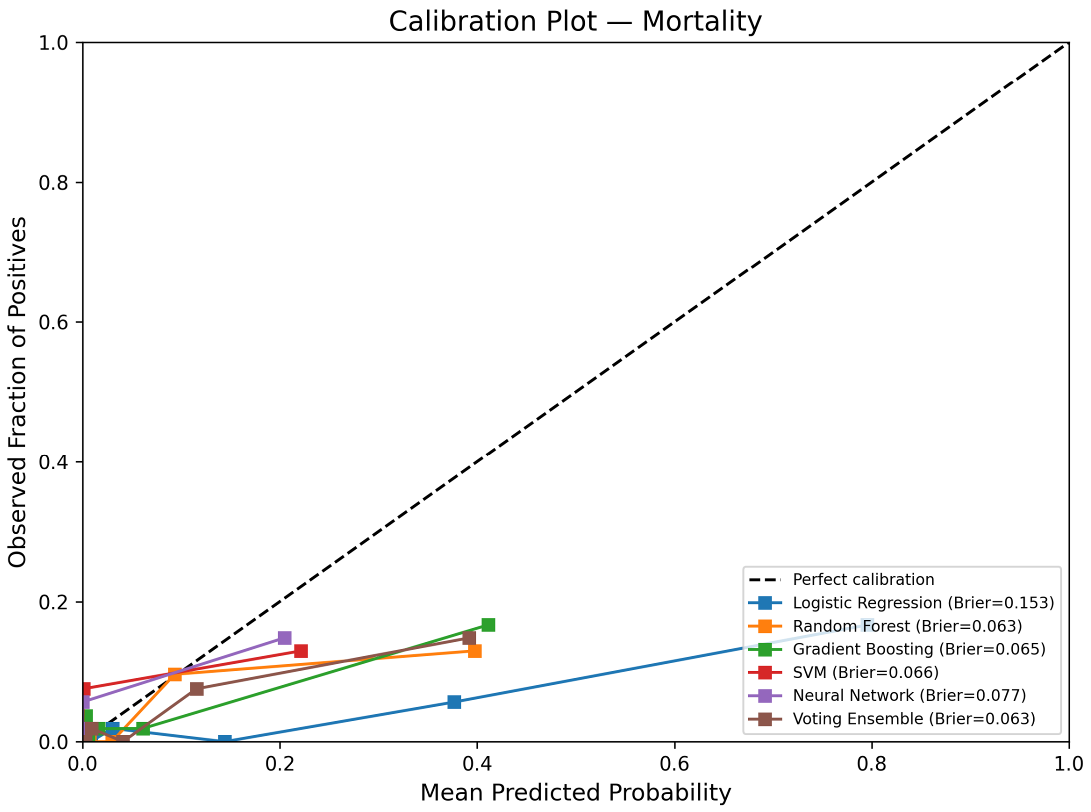
Calibration Plot — Mortality
Observed Fraction of Positives
Mean Predicted Probability
0.0
0.2
0.4
0.6
0.8
1.0
Perfect calibration
Logistic Regression (Brier=0.153)
Random Forest (Brier=0.063)
Gradient Boosting (Brier=0.065)
SVM (Brier=0.066)
Neural Network (Brier=0.077)
Voting Ensemble (Brier=0.063)

Supplementary Figure S1. Calibration plots for 90-day readmission (top) and 90-day mortality (bottom) prediction models. Calibration curves show the relationship between mean predicted probability and observed fraction of positives across quintile-based bins. The dashed diagonal line represents perfect calibration. Brier scores are reported in the legend for each model.

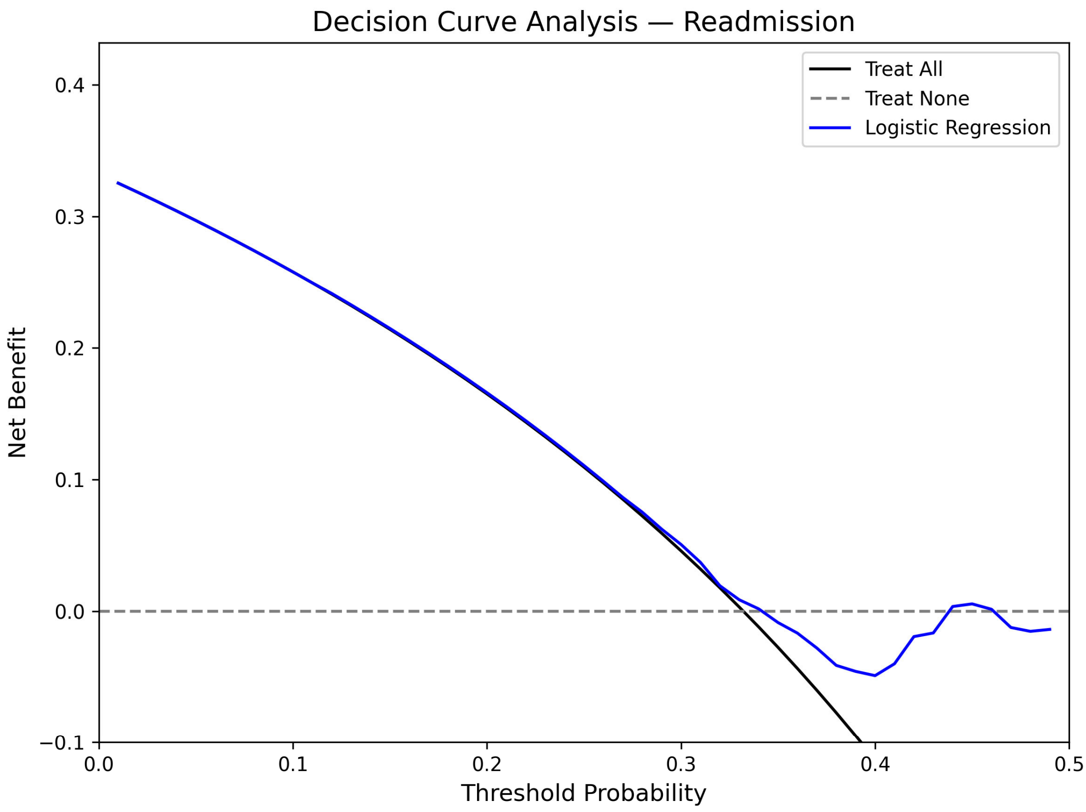
Decision Curve Analysis — Readmission
Treat All
Treat None
Logistic Regression
Net Benefit
Threshold Probability
0.4
0.3
0.2
0.1
0.0
−0.1
0.0
0.1
0.2
0.3
0.4
0.5

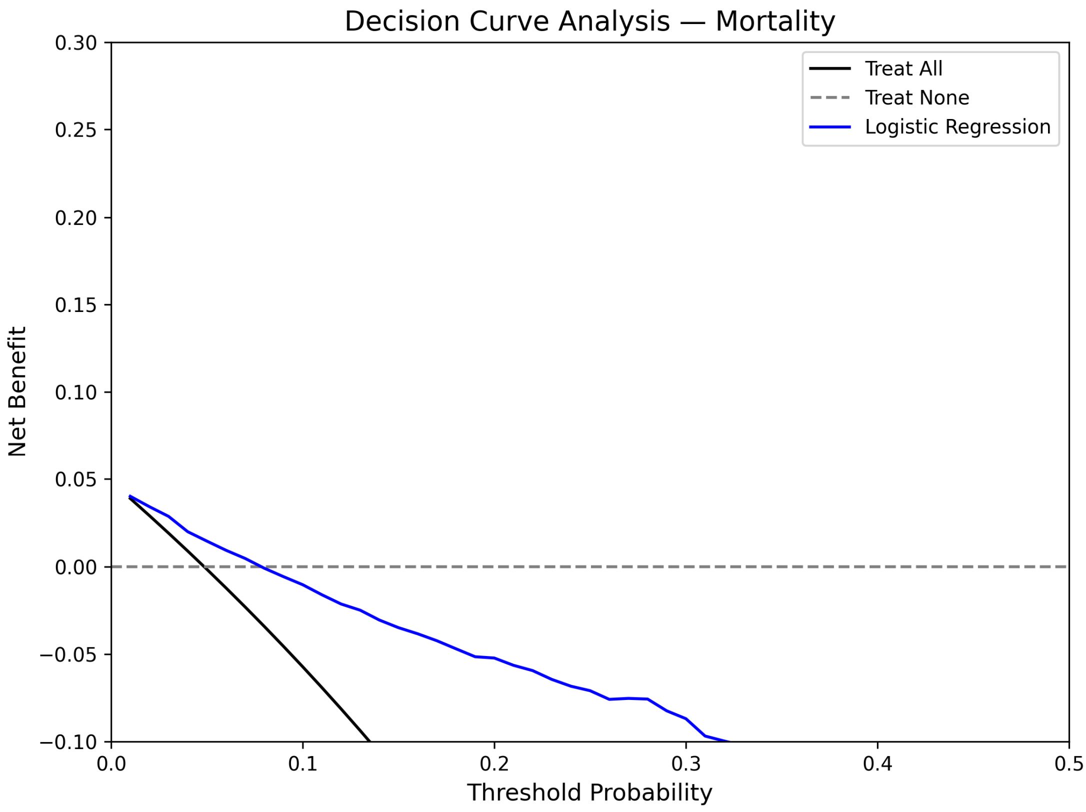
Decision Curve Analysis — Mortality
Treat All
Treat None
Logistic Regression
Net Benefit
Threshold Probability
0.30
0.25
0.20
0.15
0.10
0.05
0.00
−0.05
−0.10
0.0
0.1
0.2
0.3
0.4
0.5

Supplementary Figure S2. Decision curve analysis for 90-day readmission (top) and 90-day mortality (bottom) prediction. Net benefit is plotted against threshold probability for the best-performing model compared with treat-all and treat-none strategies. The model demonstrates net benefit over default strategies across clinically relevant threshold ranges.

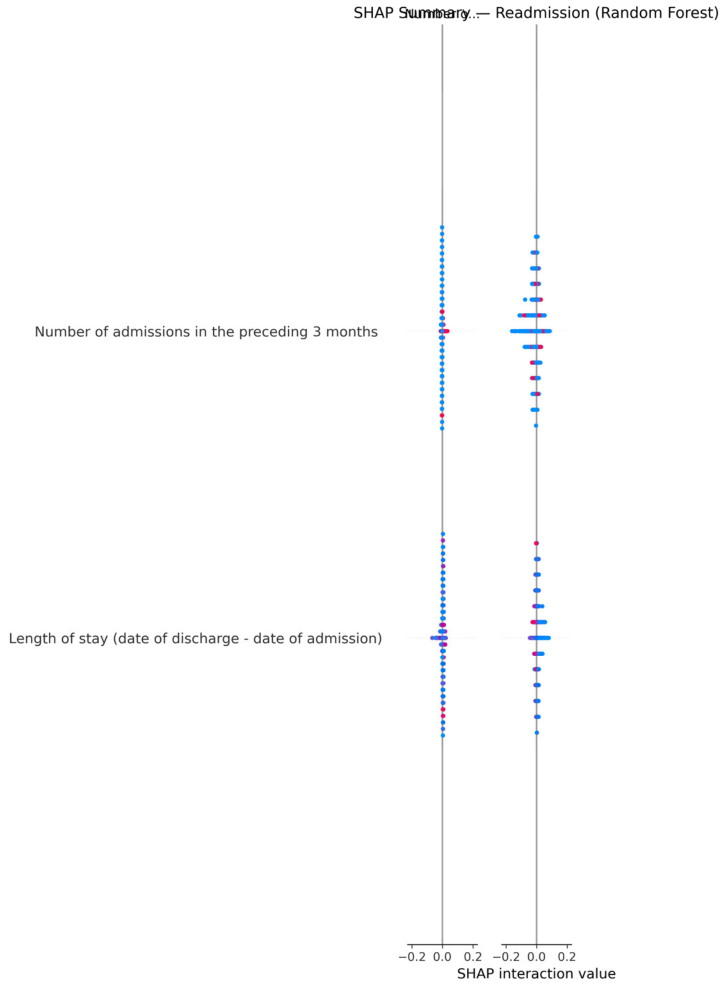
SHAP Summary — Readmission (Random Forest)
Number of admissions in the preceding 3 months
Length of stay (date of discharge - date of admission)
−0.2 0.0 0.2
−0.2 0.0 0.2
SHAP interaction value

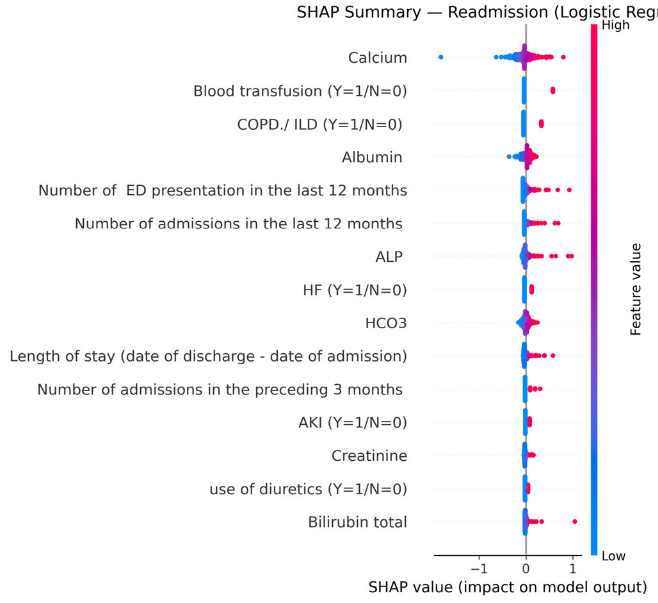
SHAP Summary — Readmission (Logistic Regression)
Calcium
Blood transfusion (Y=1/N=0)
COPD./ ILD (Y=1/N=0)
Albumin
Number of ED presentation in the last 12 months
Number of admissions in the last 12 months
ALP
HF (Y=1/N=0)
HCO3
Length of stay (date of discharge - date of admission)
Number of admissions in the preceding 3 months
AKI (Y=1/N=0)
Creatinine
use of diuretics (Y=1/N=0)
Bilirubin total
−1 0 1
SHAP value (impact on model output)
High
Low
Feature value

Supplementary Figure S3. SHAP (SHapley Additive exPlanations) summary plots for 90-day readmission prediction. Top: Random Forest model. Bottom: Logistic Regression model. Each dot represents a patient in the test set; the horizontal position indicates the SHAP value (impact on model output), and the color indicates the feature value (red = high, blue = low).

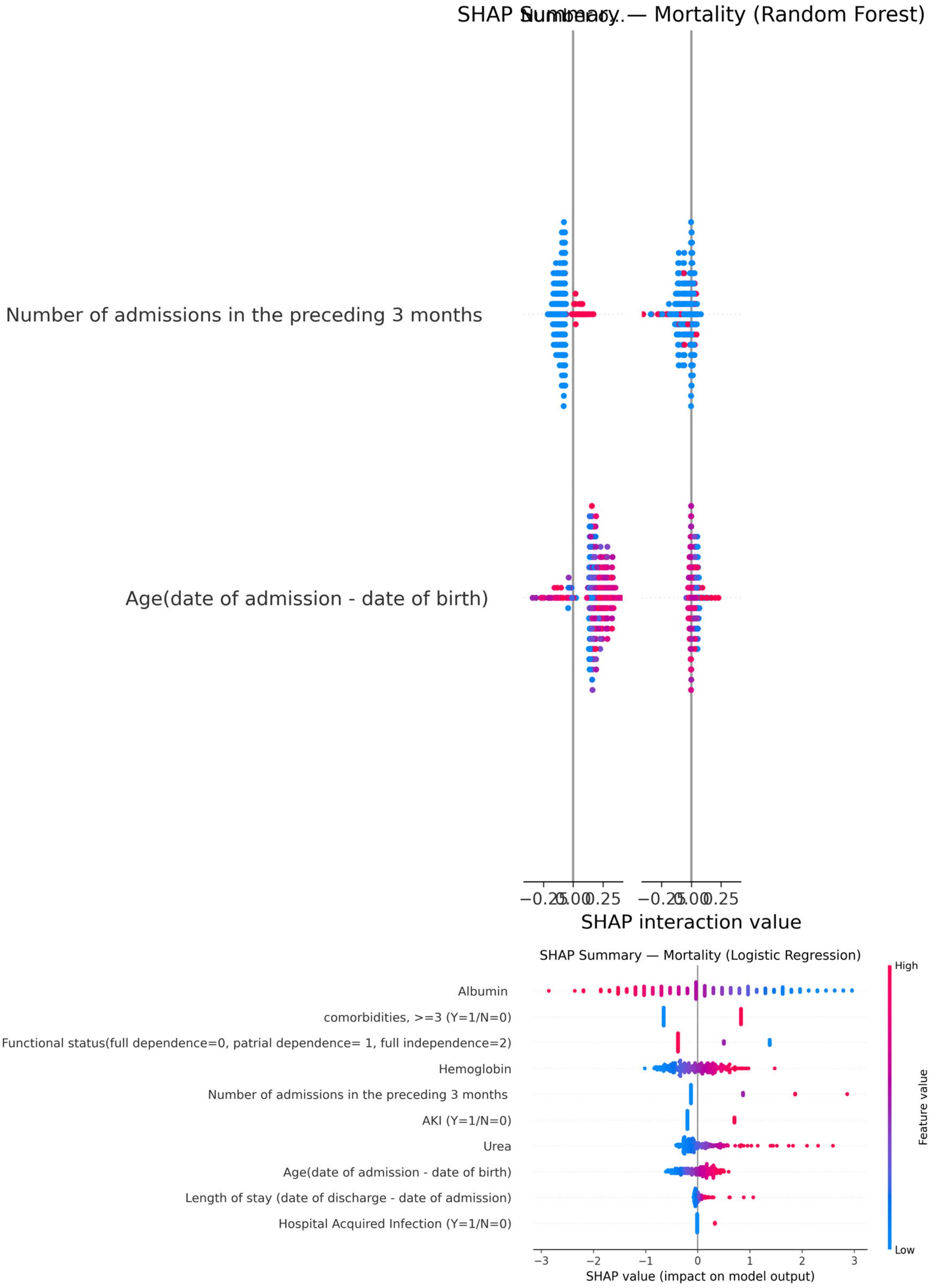

Supplementary Figure S4. SHAP (SHapley Additive exPlanations) summary plots for 90-day mortality prediction. Top: Random Forest model. Bottom: Logistic Regression model. Each dot

represents a patient in the test set; the horizontal position indicates the SHAP value (impact on model output), and the color indicates the feature value (red = high, blue = low).
