# Supplementary material for: Machine Learning Prediction of 90-Day Readmission and Mortality in General Internal Medicine: A Retrospective Cohort Study
Source: Diagnostics (Basel). 2026 Oct 14;16(20):3337. doi: 10.3390/diagnostics16203337 (PMC13703392; doi:10.3390/diagnostics16203337)
Supplement: Supplementary file 1 [file diagnostics-16-03337-s001.zip › diagnostics-4380765-supplementary tables.pdf]

Table S1. Imbalance Handling Sensitivity Analysis (AUC)

Model	Read SMOTE	Read CW	Read None	Mort SMOTE	Mort CW	Mort None
Logistic Regression	0.659	0.678	0.681	0.828	0.853	0.859
Random Forest	0.590	0.613	0.637	0.811	0.873	0.855
Gradient Boosting	0.559	0.611	0.611	0.793	0.814	0.814
SVM	0.556	0.680	0.646	0.735	0.816	0.734
Neural Network	0.539	0.535	0.535	0.776	0.797	0.797
Voting Ensemble	0.580	0.632	0.625	0.824	0.845	0.831

CW, class weighting (balanced); None, no imbalance handling; Read, readmission; Mort, mortality.

Table S2. Calibration Assessment for Primary Models

Model	Brier Score	Cal. Slope	Cal. Intercept	ECE	HL p-value
Readmission					
Logistic Regression	0.229	2.218	-1.792	0.156	<0.001
Random Forest	0.223	1.353	-1.227	0.086	0.039
Gradient Boosting	0.239	0.970	-1.071	0.147	<0.001
SVM	0.250	0.702	-0.992	0.158	<0.001
Neural Network	0.372	0.219	-0.764	0.343	<0.001
Voting Ensemble	0.230	1.153	-1.156	0.119	<0.001
Mortality					
Logistic Regression	0.127	2.146	-3.678	0.190	<0.001
Random Forest	0.059	1.654	-3.159	0.072	<0.001
Gradient Boosting	0.060	1.682	-3.153	0.056	<0.001

Voting Ensemble	0.060	1.995	-3.257	0.062	<0.001

ECE, expected calibration error; HL, Hosmer–Lemeshow; Cal., calibration.

Table S3. Feature Count (k) Sensitivity Analysis

Configuration	Best Model	AUC (95% CI)	EPV
Readmission k=20	LR	0.659 (0.584-0.729)	14.7
Readmission k=15	LR	0.675 (0.602-0.745)	19.6
Mortality k=10	LR	0.828 (0.694-0.930)	4.2
Mortality k=5	LR	0.807 (0.651-0.938)	8.4

EPV, events per variable; LR, Logistic Regression.

Table S4. Temporal Validation (Train 2022, Test 2023)

Model	Readmission AUC (95% CI)	Mortality AUC (95% CI)
Train set	479 patients (201 events)	479 patients (29 events)
Test set	402 patients (93 events)	402 patients (13 events)
Logistic Regression	0.673 (0.603-0.738)	0.891 (0.825-0.940)
Random Forest	0.614 (0.550-0.675)	0.854 (0.757-0.925)
Gradient Boosting	0.596 (0.519-0.665)	0.833 (0.756-0.901)
Neural Network	0.580 (0.505-0.649)	0.861 (0.799-0.915)
Random-split (primary)	0.659 (0.584-0.729)	0.828 (0.694-0.930)

Table S5. Best Hyperparameters from Grid Search

Model	Readmission	Mortality
Logistic Regression	C=0.01	C=0.1
Random Forest	max_depth=None, n_est=200	max_depth=None, n_est=100
Gradient Boosting	lr=0.1, n_est=100	lr=0.1, n_est=100
SVM	C=10, gamma=scale	C=10, gamma=scale
Neural Network	layers=(100,50)	layers=(100,50)

n_est, n_estimators; lr, learning_rate; layers, hidden_layer_sizes.

Table S6. DNR Sensitivity Analysis — Mortality Models

Model	With DNR (AUC)	Without DNR (AUC)	Delta
Logistic Regression	0.828	0.823	−0.005
Random Forest	0.811	0.788	−0.022

Gradient Boosting	0.793	0.759	−0.034
SVM	0.735	0.779	+0.044
Neural Network	0.776	0.816	+0.040
Voting Ensemble	0.824	0.821	−0.003

Table S7. Feature Selection Method Comparison

Outcome	Method	Features Selected	Overlap	LR AUC
Readmission	SelectKBest vs LASSO	20 vs 10	10/10 (100%)	0.659 vs 0.671
Mortality	SelectKBest vs LASSO	10 vs 13	7/10 (70%)	0.828 vs 0.757
